# Diagnostic accuracy of an enzyme-based point-of-care test versus Nugent score for bacterial vaginosis among pregnant women attending routine antenatal care in Zambia

**DOI:** 10.1186/s12879-026-12714-y

**Published:** 2026-01-31

**Authors:** Sumire Sorano, Enesia Banda Chaponda, Massimo Mirandola, Ephraim Chikwanda, Vivian Mwewa, Joyce M. Mulenga, Mike Chaponda, Ludovica Ghilardi, Emma M. Harding-Esch, Chris Smith, Mitsuaki Matsui, Daniel Chandramohan, Mohamed Mahmoud Ali, Karel Blondeel, Magnus Unemo, Igor Toskin, R. Matthew Chico

**Affiliations:** 1 Faculty of Infectious and Tropical Diseases, London School of Hygiene & Tropical Medicine, London, WC1E 7HT UK; 2 School of Tropical Medicine and Global Health, Nagasaki University, 1-14 Bunkyomachi, Nagasaki, 852-8521 Japan; 3 Department of Biosciences and Biotechnology, University of Zambia, Lusaka, Zambia; 4 Department of Diagnostics and Public Health, University of Verona, Verona, Italy; 5 National Health Research and Training Institute, Ndola, Zambia; 6 St. Paul’s Mission Hospital, Nchelenge, Zambia; 7 WHO Collaborating Centre for Sexually Transmitted Infections, London School of Hygiene & Tropical Medicine, London, UK; 8 Division of Global Health, Department of Public Health, Graduate School of Health Sciences, Kobe University, Kobe, Japan; 9 Department of Sexual and Reproductive Health and Research, World Health Organization, Geneva, Switzerland; 10 Faculty of Medicine and Health Sciences, Ghent University, Ghent, Belgium; 11 Department of Laboratory Medicine, Faculty of Medicine and Health, WHO Collaborating Centre for Gonorrhoea and Other STIs, Örebro University, Örebro, Sweden; 12 Institute for Global Health, University College London (UCL), London, UK

**Keywords:** Bacterial vaginosis, Point-of-care test, OSOM BVBlue, Nugent score, Diagnostic test accuracy, Pregnancy, Antenatal care (ANC)

## Abstract

**Background:**

Bacterial vaginosis (BV) is associated with adverse pregnancy outcomes. OSOM^®^ BVBlue^®^ is a chromogenic point-of-care (POC) test that detects sialidase, an enzyme produced by *Gardnerella vaginalis* and some other anaerobic bacteria associated with BV. This study, part of the World Health Organization’s global ProSPeRo study, aimed to evaluate the performance of this POC test compared with the Nugent score reference standard among pregnant women in Zambia. Additionally, the operational characteristics and patient acceptability of the POC test were evaluated.

**Methods:**

Pregnant women attending four health centres in Nchelenge, Zambia, for antenatal care between 15 February and 26 May 2023 participated. Clinician-collected vaginal swabs for OSOM^®^ BVBlue^®^ and Nugent scoring were obtained from each participant. POC test results were read independently by two staff members. Study staff completed a questionnaire on the operational characteristics of the POC test, whereas participants were asked about the length of time that they would be willing to wait for POC test results.

**Results:**

Paired POC and reference test vaginal swabs from 999 participants were analysed. Overall, 23.1% (231/999) tested positive for BV by Nugent score. Overall sensitivity and specificity of OSOM^®^ BVBlue^®^ were 41.3% (95% confidence interval [CI] 25.4–59.2%) and 93.1% (95% CI 89.8–95.4%), respectively. Inter-rater agreement was 99.6% (Cohen’s Kappa 0.984). Most pregnant women, 97.9% (978/999), were willing to wait up to 20 min for a POC test result, and 59.2% (591/999) up to 30 min. Of 14 study staff members, all found the POC test easy to use and instructions clear, 13 felt the results were easy to interpret, with 12 reporting test results were available within 25 min. Cold-chain requirements and short shelf life were perceived as significant challenges.

**Conclusion:**

Overall, OSOM^®^ BVBlue^®^ yielded sub-optimal sensitivity. The findings from this study are valuable for the development of minimal and optimal product profiles for POC tests for BV. These profiles, in turn, may guide future research and development for this prevalent condition with important sexual and reproductive health consequences. Logistical challenges need to be addressed for effective implementation of POC testing in resource-limited settings.

**Trial registration:**

PACTR202302766902029.

**Supplementary Information:**

The online version contains supplementary material available at 10.1186/s12879-026-12714-y.

## Introduction

Bacterial vaginosis (BV), the most common urogenital condition in the world, is a vaginal dysbiosis that results from a depletion of commensal *Lactobacillus* species and an overgrowth of anaerobic bacteria [1, 2]. A recent systematic review and meta-analysis estimated that 25.1% (95% CI: 17.5–33.6%; *N* = 23,028) of pregnant women have BV in sub-Saharan Africa [3]. BV is associated with an increased risk of preterm delivery and spontaneous abortion [4], postpartum endometritis, and the acquisition and transmission of HIV and several other sexually transmitted infections (STIs) [5–7]. BV has also been associated with Human Papillomavirus infection and subsequent cervical pathology [8–10].

Despite these associations between BV and adverse pregnancy outcomes, screening and treatment of asymptomatic BV during pregnancy remain controversial. Meta-analyses of randomised controlled trials suggest that treating asymptomatic BV does not significantly lower the rates of preterm birth [11]. However, certain groups of women, especially those at higher risk for preterm birth, might benefit from treatment [12, 13].

Symptoms of BV may vary; some individuals remain asymptomatic, whereas others may experience thin, grey or white vaginal discharge with a strong fishy odor, itching, or burning during urination. Diagnosing BV in resource-limited settings is a challenge [14]. Use of Nugent scores, which involves microscopic examination of a vaginal swab, is considered the “gold standard” for laboratory diagnosis of BV, but it requires laboratory expertise that is uncommon in many resource-limited settings [15]. Syndromic-based diagnosis and treatment algorithms have been developed to aid the management of symptomatic cases of vaginal discharge in these settings [16]. However, because BV symptoms are non-specific and many affected women are asymptomatic, this approach cannot effectively identify all BV cases. In antenatal care, the sensitivity of these algorithms for treating BV among women with vaginal discharge ranges from 13% to 50%, whereas specificity spans 54% to 84% [17–19].

Point-of-care (POC) tests for BV are available and may facilitate rapid antenatal case-detection and treatment [15]. OSOM^®^ BVBlue^®^ (Sekisui Diagnostics, LLC, Massachusetts, USA) POC tests have a Clinical Laboratory Improvement Amendments (CLIA) waiver from the United States Food and Drug Administration, and have been used in clinics as part of sexual and reproductive services among women presenting with symptoms associated with BV. However, there is a lack of research evaluating this POC test for screening pregnant women. As part of the World Health Organization’s (WHO) global ProSPeRo study (Project on STI POC Testing), the primary objective of our study was to evaluate the clinical performance of an enzyme-based point-of-care technology in an antenatal care (ANC) setting in Zambia using OSOM^®^ BVBlue as an index test. We also assessed the operational characteristics and acceptability of this technology for patients and healthcare providers.

## Methods

We registered this evaluation in the Pan African Clinical Trials Registry (PACTR202302766902029, dated 13/09/2022) and prepared this report in accordance with the Standard for Reporting Diagnostic Accuracy (STARD) guidelines [20]. We evaluated the performance of OSOM^®^ BVBlue^®^ compared with the Nugent score reference standard (≥ 7 indicating BV) for diagnosis of BV in pregnant women in rural Zambia. The study also evaluated the performance of an antigen-based POC test for trichomoniasis, the results of which have been reported elsewhere [21]. In brief, we present here the methods that are specific to the evaluation of OSOM^®^ BVBlue^®^. OSOM^®^ BVBlue^®^ is an enzyme-based POC test that includes a chromogenic substrate to detect sialidase, an enzyme produced by bacterial pathogens associated with BV, including *Gardnerella vaginalis*, *Bacteroides*, *Prevotella* and *Mobiluncus*. The POC test turns the sample swab blue or green in the presence of high levels of sialidase, whereas yellow indicates a negative result (absence of sialidase). Nugent scoring, the reference standard used in our study, is based on the presence of three bacteria morphotypes: *Lactobacillus*, *Gardnerella*, and *Mobiluncus*. It is the most commonly used diagnostic method in research settings, allowing for slides to be preserved and revisited, providing a quantitative analysis.

### Study site

The study was conducted in four health centres that provide ANC in the Nchelenge District in Zambia: Kabuta, Kafutuma, Kashikishi, and Nchelenge. All centres were within the catchment area of Saint Paul’s Mission Hospital in Luapula Province, located in northern Zambia along the eastern shore of Lake Mweru [22].

### Study participants

We recruited women attending ANC who were pregnant for no less than 13 gestational weeks and self-reported having: (i) not received metronidazole or clindamycin during their current pregnancy; (ii) not having a known allergy or other contraindication to metronidazole, and (iii) not used a vaginal cream or ointment product, douched or used vaginal lubricants within 72 h of recruitment. Pregnant women of all ages were eligible. Consistent with Zambian law, if potential participants were under 18 years of age, they would provide assent followed by their guardians consenting.

### Procedures

The study design was aligned with the daily clinical practice of the study sites. All women who attended antenatal care were invited to participate until the target sample size was met. After obtaining written informed consent, we measured gestational age by sonography. For eligible participants, background and clinical information were collected by the study staff through participant interviews and antenatal records. Questions included self-reported vaginal symptoms (unusual vaginal discharge, pain during urination, itching or burning of the vulva) and other STI-related symptoms (sores, blisters, ulcers, warts or rashes in the genital area, lumps and bumps on the genitals, and lower abdominal pain). HIV and syphilis testing was conducted as part of routine ANC. HIV status or HIV/syphilis test results were obtained from the antenatal records. Additionally, we inquired about the patients’ willingness to wait for POC test results. This information was recorded in an electronic case report form designed in REDCap software [23].

Clinical staff collected four vaginal swabs from participants, one for each of the four assays: BV POC test, TV POC test (results reported elsewhere), Nugent scoring, and TV nucleic acid amplification test (NAAT) analysis (results reported elsewhere). The order of swab collection was randomised to prevent any bias related to the quantity of the vaginal sample that could arise from the sequence of collection. Considering that there were four swabs to be collected, there were 24 variations possible for sample collection. Prior to study initiation, we generated a random list of 1,100 sampling sequences. An independent researcher, not otherwise involved in the study, prepared opaque envelopes containing the sample collection order and participant IDs. Once participants were deemed eligible, they opened an envelope to reveal the swab order. For Nugent scoring, swabs were immediately rolled onto a glass slide after collection. POC test samples were promptly transported to a designated area and processed by clinical or laboratory staff in accordance with the manufacturer’s instructions. Results were independently read by two clinicians, or one clinician and one laboratory staff member, with each entering the results into REDCap sequentially without knowing the other’s reading. A separate study staff member then communicated the POC test results to participants in writing. If participants tested positive on the POC test based on at least one of the two readings, the study staff administered a 2-gram dose of metronidazole during the same visit, a recommended curative treatment for BV. To ensure that study staff used the POC tests as designed, we involved representatives of the POC test manufacturer in our onsite Investigators’ Meeting and training. The manufacturer had no further involvement. However, throughout the conduct of the study, laboratory technicians used a quality control kit as contained in each new box of OSOM^®^ BVBlue^®^ POC tests. At the end of the study, we provided a questionnaire to the clinic staff to evaluate the operational characteristics of the POC tests.

### Laboratory analysis

Glass slide samples for Nugent scoring were transported to Saint Paul’s Mission Hospital where Gram staining was performed on the same day as collection. The Gram-stained slides were then sent to the Tropical Diseases Research Centre in Ndola, Zambia, (renamed as the National Health Research and Training Institute) where two laboratory staff members independently read them for Nugent scoring, blinded to all clinical information and POC test results. HIV and syphilis tests were conducted as part of routine antenatal care. Core tests^®^ ONE STEP Syphilis Test Kits (Core Technology, Atlanta, USA) were used for syphilis screening, and Determine^®^ HIV Test Kits (Abbott, Illinois, USA) were used for HIV screening.

### Statistical analysis

We compared the POC test results (positive, negative, indeterminate, or invalid) against Nugent scores. Any indeterminate or invalid results, as well as instances of missing data, were excluded from the diagnostic accuracy analysis measures; we reported the number of invalid results separately. Pooled sensitivity, specificity, positive and negative likelihood ratios (LR), and positive (PPV) and negative predictive values (NPV) were estimated using a bivariate random effect meta-analysis model with logit transformation [24] which took into account the correlation between sensitivity and specificity. The model addresses both within-site variation and between-site variations. Stata midas and metadta packages [25–27] were used for the meta-analysis. Sub-group analyses were conducted based on presence of BV-associated symptoms. Additionally, PPV and NPV were estimated based on prevalence scenarios derived from study point estimates and 95% confidence intervals, a method used in other ProSPeRo studies [21]. Positive and negative LRs were plotted using Fagan’s nomogram in order to obtain post-test disease probability. Observed inter-rater agreement and Cohen’s Kappa were calculated. Additionally, we examined the relationship between Nugent scores and POC test positivity using descriptive statistics. To determine the specific bacterial morphotypes corresponding with BVBlue^®^ positivity, we employed univariate and multivariate logistic regression analysis. We used Stata 18.0 software [28] for our analyses.

**Fig. 1:**
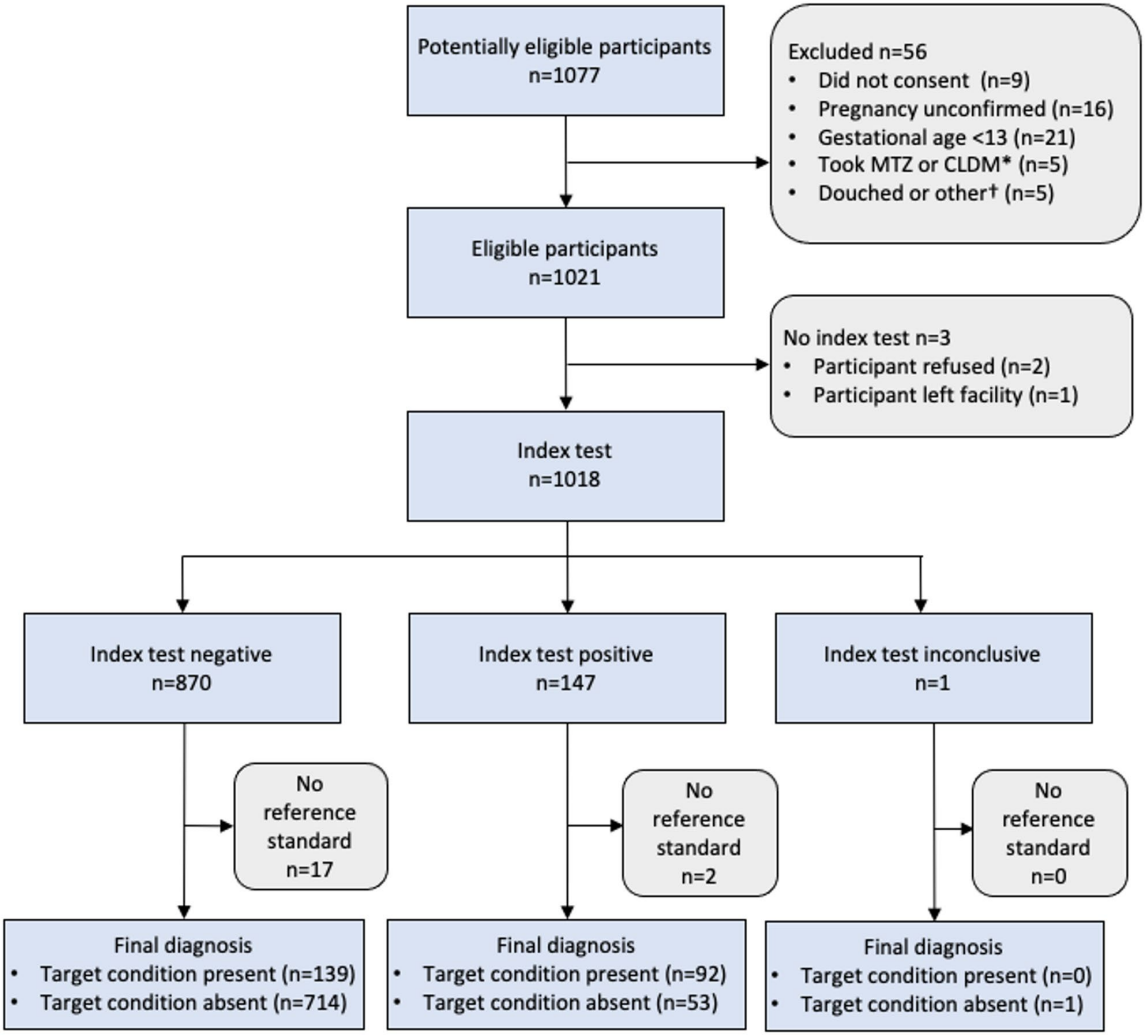
STARD flow diagram. *Metronidazole or clindamycin use during current pregnancy; †any of the following within 72 h of visit: use of vaginal cream or ointment, spermicides, vaginal lubricants or feminine sprays, or douching

## Results

We screened 1,077 potentially eligible pregnant women and enrolled 1,021 of them between February 15 and May 26, 2023. Three participants refused swab collection for both or either of the POC and reference test, and 19 glass slides were damaged accidentally in the laboratory prior to analysis. Ultimately, we obtained BV POC test results and Nugent scores from 999 women (Fig. 1). The majority of participants were aged 18–24 years with an age range of 14–46 years (Table 1). The majority of pregnant women, 71.0%, were married or living with a partner, whereas remaining participants, 29.0%, were single, separated, divorced, or widowed. In total, 56.4% of women were in their second trimester (13–27 gestational weeks), whereas 43.6% were in their third trimester (28–39 gestational weeks). Additionally, 8.7% were HIV sero-positive, and 14.9% were sero-positive for syphilis.

**Table 1:** Basic characteristics and clinical variables of participating pregnant women in Nchelenge, Zambia

Basic characteristics (*N* = 999)	Number	Percentage
Facility		
Kabuta	138	13.8%
Kafutuma	143	14.3%
Kashikishi	436	43.6%
Nchelenge	282	28.2%
Age group (years)		
14–17	35	3.5%
18–24	542	54.3%
25–34	319	31.9%
35–46	103	10.3%
Marital status		
Single	280	28.1%
Married or living with a partner	709	71.0%
Separated, divorced or widowed	9	0.9%
Number of previous pregnancies		
0	301	30.1%
1	222	22.2%
2	177	17.7%
3+	299	29.9%
Gestational age (in weeks)		
13–27	563	56.4%
28–39	436	43.6%
Symptoms associated with BV		
Unusual vaginal discharge*	62	6.2%
Pain during urination*	43	4.3%
Itching or burning of the vulva*	153	15.3%
At least one symptom above†	193	19.4%
HIV status		
Positive	87	8.7%
Negative	902	90.3%
Unknown	10	1.0%
Syphilis point-of-care test		
Positive	149	14.9%
Negative	846	84.7%
Unknown	4	0.4%

*One participant was missing a value for this symptom

†Two participants were missing values for at least one symptom

The POC test results and Nugent scores by facility are provided in Additional file 1 Table S2. Table 2 shows prevalence estimates based on Nugent score and diagnostic accuracy measures of the OSOM^®^ BVBlue^®^ compared with Nugent score. BV prevalence estimates ranged from 21.8% in Kafutuma to 26.1% in Kabuta. One sample produced indeterminate or invalid POC test results which was negative by Nugent score.

**Fig. 2:**
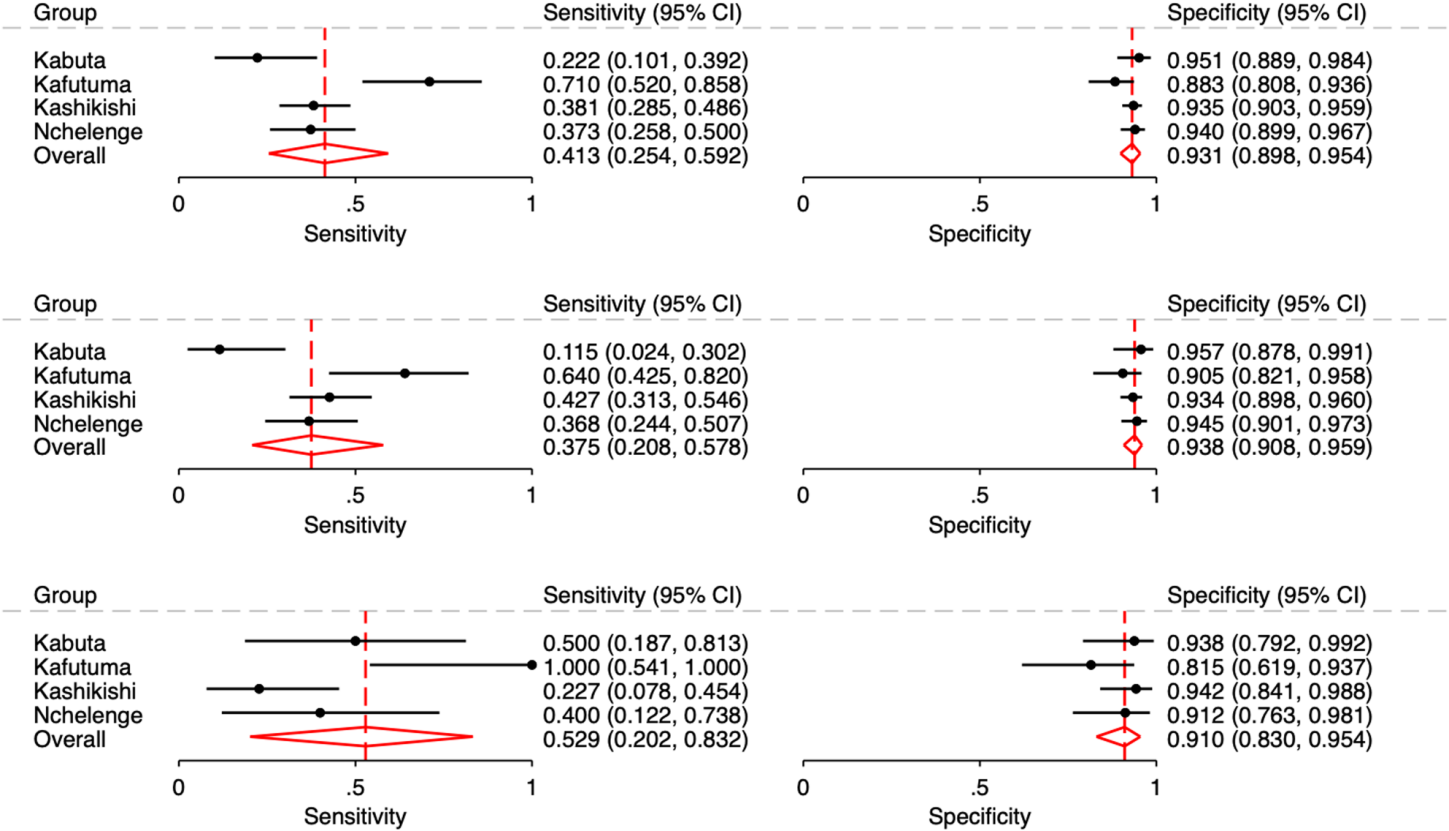
Forest plots of sensitivity and specificity of OSOM^®^ BVBlue^®^ compared to Nugent score for bacterial vaginosis among pregnant women attending antenatal care by health facility and overall in Nchelenge, Zambia

Figure 2 shows forest plots of sensitivity and specificity of the POC test, overall and by presence of symptoms. Sensitivity ranged from 22.2% in Kabuta to 71.0% in Kafutuma, and specificity ranged from 88.3% in Kafutuma to 95.1% in Kabuta. Pooled prevalence, sensitivity and specificity across facilities were 23.1% (95% CI 20.5–26.2%), 41.3% (95% CI 25.4–59.2%) and 93.1% (95% CI 89.8–95.4%), respectively. There was no evidence of a statistical difference in prevalence between individuals with and without BV-associated symptoms (24.4% [95%CI 18.3–30.4] versus 22.7% [95%CI 19.8–25.5]; relative ratio [RR] 1.08, 95% CI 0.78–1.48; *P* = 0.645). Similarly, there was no evidence of a significant difference in sensitivity between pregnant women with and without BV-related symptoms (52.9% [95%CI 20.2–83.2] versus 37.5% [95%CI 20.8–57.8], RR 1.41, 95% CI 0.69–2.87; *P* = 0.342). Table 3 shows PPV and NPV of the BV POC test based on prevalence scenarios of the minimum, maximum, and pooled prevalence of the study sites. Overall, PPV ranged from 61.5% to 67.8%, and NPV ranged from 81.9% to 85.7%. Fagan’s nomogram, plotted based on prevalence scenario and positive and negative LR (Additional file 1 Figure S1), illustrates these results. Observed inter-rater agreement of POC test results was 99.6% (995/999), with a Cohen’s Kappa of 0.984.

**Table 2:** Performance of OSOM^®^ BVBlue^®^ compared to Nugent score for bacterial vaginosis among pregnant women attending antenatal care overall and among asymptomatic and symptomatic participants

Participant characteristics	*N*	Prevalence (%) (95% CI)†	Sensitivity (%) (95% CI)	Specificity (%) (95% CI)
**Overall (symptomatic and asymptomatic participants)**				
Kabuta	138*	26.1 (19.0–34.2)	22.2 (10.1–39.2)	95.1 (88.9–98.4)
Kafutuma	142	21.8 (15.3–29.5)	71.0 (52.0–85.8)	88.3 (80.8–93.6)
Kashikishi	436*	22.2 (18.4–26.4)	38.1 (28.5–48.6)	93.5 (90.3–95.9)
Nchelenge	282	23.8 (18.9–29.2)	37.3 (25.8–50.0)	94.0 (89.9–96.7)
Pooled estimate	998*	23.1 (20.5–26.2)	41.3 (25.4–59.2)	93.1 (89.8–95.4)
Positive likelihood ratio = 6.0 (4.3–8.3)				
Negative likelihood ratio = 0.63 (0.48–0.83)				
Diagnostic odds ratio = 10 (6–16)				
**Asymptomatic participants**				
Kabuta	95*	27.4 (18.7–37.5)	11.5 (2.4–30.2)	95.7 (87.8–99.1)
Kafutuma	109	22.9 (15.4–32.0)	64.0 (42.5–82.0)	90.5 (82.1–95.8)
Kashikishi	361*	20.8 (16.7–25.3)	42.7 (31.3–54.6)	93.4 (89.8–96.0)
Nchelenge	238	23.9 (18.7–29.9)	36.8 (24.4–50.7)	94.5 (90.1–97.3)
Pooled estimate	803*	22.7 (19.8–25.5)	37.5 (20.8–57.8)	93.8 (90.8–95.9)
Positive likelihood ratio = 6.1 (3.9–9.4)				
Negative likelihood ratio = 0.67 (0.50–0.89)				
Diagnostic odds ratio = 9 (5–18)				
**Symptomatic participants**				
Kabuta	42*	23.8 (12.1–39.5)	50.0 (18.7–81.3)	93.8 (79.2–99.2)
Kafutuma	33	18.2 (7.0–35.5)	100 (54.1–100.0)	81.5 (61.9–93.7)
Kashikishi	74*	29.7 (19.7–41.5)	22.7 (7.8–45.4)	94.2 (84.1–98.8)
Nchelenge	44	22.7 (11.5–37.8)	40.0 (20.2–83.2)	91.2 (76.3–98.1)
Pooled estimate	193*	24.4 (18.3–30.4)	52.9 (20.2–83.2)	91.0 (83.0–95.4)
Positive likelihood ratio = 5.9 (2.9–11.7)				
Negative likelihood ratio = 0.52 (0.24–1.10)				
Diagnostic odds ratio = 11 (3–42)				

Symptomatic participants had at least one of the following: unusual vaginal discharge, pain during urination, itching or burning of the vulva; *The sum of symptomatic and asymptomatic cases is not equal to the overall count; one case each in Kabuta and Kashikishi was missing symptom information

†Prevalence is based on reference method. CI: confidence interval

**Table 3:** Positive predictive value and negative predictive value of OSOM^®^ BVBlue^®^ based on prevalence scenarios among pregnant women attending antenatal care in Nchelenge, Zambia

Prevalence range (%)*	Pooled sensitivity (%)	Pooled specificity (%)	Prevalence scenarios	Positive predictive value (95%CI)	Negative predictive value (95%CI)
**Overall (symptomatic and asymptomatic participants)**					
23 (21–26)	41.3	93.1	21	61.5 (53.6–68.8)	85.7 (81.9–88.8)
			23	64.2 (56.5–71.2)	84.2 (80.1–87.5)
			26	67.8 (60.4–74.4)	81.9 (77.3–85.7)
**Asymptomatic participants**					
23 (20–26)	37.5	93.8	20	60.3 (49.6–70.1)	85.7 (81.7–89.0)
			23	64.4 (54.0–73.7)	83.4 (78.9–87.1)
			26	68.1 (58.0–76.7)	81.0 (76.1–85.2)
**Symptomatic participants**					
24 (18–30)	52.9	91.0	18	56.3 (39.2–72.0)	89.8 (80.5–94.9)
			24	65.0 (48.1–78.7)	85.9 (74.1–92.9)
			30	71.6 (55.8–83.4)	81.8 (67.9–90.6)

*Prevalence is based on reference method; 95%CI: 95% confidence interval; Prevalence scenarios are based on the minimum and maximum of the observed site and pooled prevalence

**Fig. 3:**
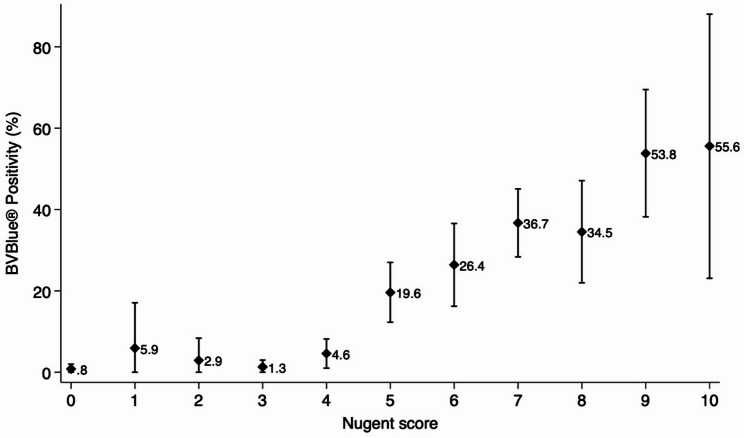
Association of OSOM^®^ BVBlue^®^ positivity and Nugent score among pregnant women attending antenatal care in Nchelenge, Zambia

Figure 3 shows the relationship between OSOM^®^ BVBlue^®^ positivity and Nugent scores,. The graph illustrates that BVBlue^®^ positivity increases with higher Nugent scores (0.8% [95%CI 0.0, 2.0%] for score 0, 19.6% [95%CI 12.3, 27.0] for score 5, 55.6% [95%CI 23.1, 88.8] for score 10). We analysed which bacterial morphotypes of Nugent scoring corresponded with BVBlue^®^ positivity. Table 4 presents the results of OSOM^®^ BVBlue^®^ positivity in relation to scores for each bacterial category. To differentiate the positive or negative correlation of these three bacterial categories being present, we employed multivariate logistic regression models to generate an adjusted odds ratio (aOR) that reflected a score-response relationship between *Lactobacillus* score and OSOM^®^ BVBlue^®^ positivity. Even a mild reduction of *Lactobacillus* (score 1 = 5–30 per field) was associated with increased OSOM^®^ BVBlue^®^ positivity (aOR 3.25 [95%CI 1.58, 6.66], p value = 0.001), with a more substantial increase observed in the absence of *Lactobacillus* (score 4: aOR 11.40 [95%CI 4.26, 30.48], p value = < 0.001). Conversely, there was no evidence of a significant increase of OSOM^®^ BVBlue^®^ positivity with a mild presence of *Gardnerella* (score 3 = 5–30 per field: aOR 2.25 [95%CI 0.59, 10.12], p value = 0.22), but increased sharply when *Gardnerella* was abundant (score 4 = > 30 per field: aOR8.47 [95%CI 2.47, 29.02], p value = 0.001). The presence of *Mobiluncus* showed an association with OSOM^®^ BVBlue^®^ positivity in the univariate analysis. However, this relationship was no longer detectable after adjusting with *Lactobacillus* and *Gardnerella* scores.

**Table 4:** Association of OSOM^®^ BVBlue^®^ positivity and subcategories of Nugent scores among pregnant women attending antenatal care in Nchelenge, Zambia

	*N*	POCT positive (%)	Unadjusted OR	95% CI	*p* value	Adjusted OR	95% CI	*p* value
*Lactobacillus* Score								
0	552	14 (2.5%)	1			1		
1	178	30 (16.9%)	**7.79**	**4.03**,** 15.07**	**< 0.001**	**3.25**	**1.58**,** 6.66**	**0.001**
2	101	27 (26.7%)	**14.02**	**7.03**,** 27.95**	**< 0.001**	**4.95**	**2.30**,** 10.64**	**< 0.001**
3	139	62 (44.6%)	**30.94**	**16.52**,** 57.94**	**< 0.001**	**11.67**	**5.82**,** 23.40**	**< 0.001**
4	28	12 (42.9%)	**28.82**	**11.51**,** 72.14**	**< 0.001**	**11.40**	**4.26**,** 30.48**	**< 0.001**
*Gardnerella* Score								
0	263	3 (1.1%)	1			1		
1	7	0 (0.0%)	1			1		
2	42	1 (2.4%)	2.11	0.21, 20.81	0.52	1.25	0.12, 12.89	0.85
3	182	6 (3.3%)	2.95	0.73, 11.97	0.13	2.25	0.59, 10.12	0.22
4	504	135 (26.8%)	**31.7**	**9.99**,** 100.64**	**< 0.001**	**8.47**	**2.47**,** 29.02**	**0.001**
*Mobiluncus* Score								
0	851	94 (11.0%)	1					
1	10	4 (40.0%)	**5.37**	**1.49**,** 19.37**	**0.01**	1.39	0.36, 5.42	0.635
2	137	47 (34.3%)	**4.21**	**2.78**,** 6.35**	**< 0.001**	1.36	0.86, 2.16	0.184

**Fig. 4:**
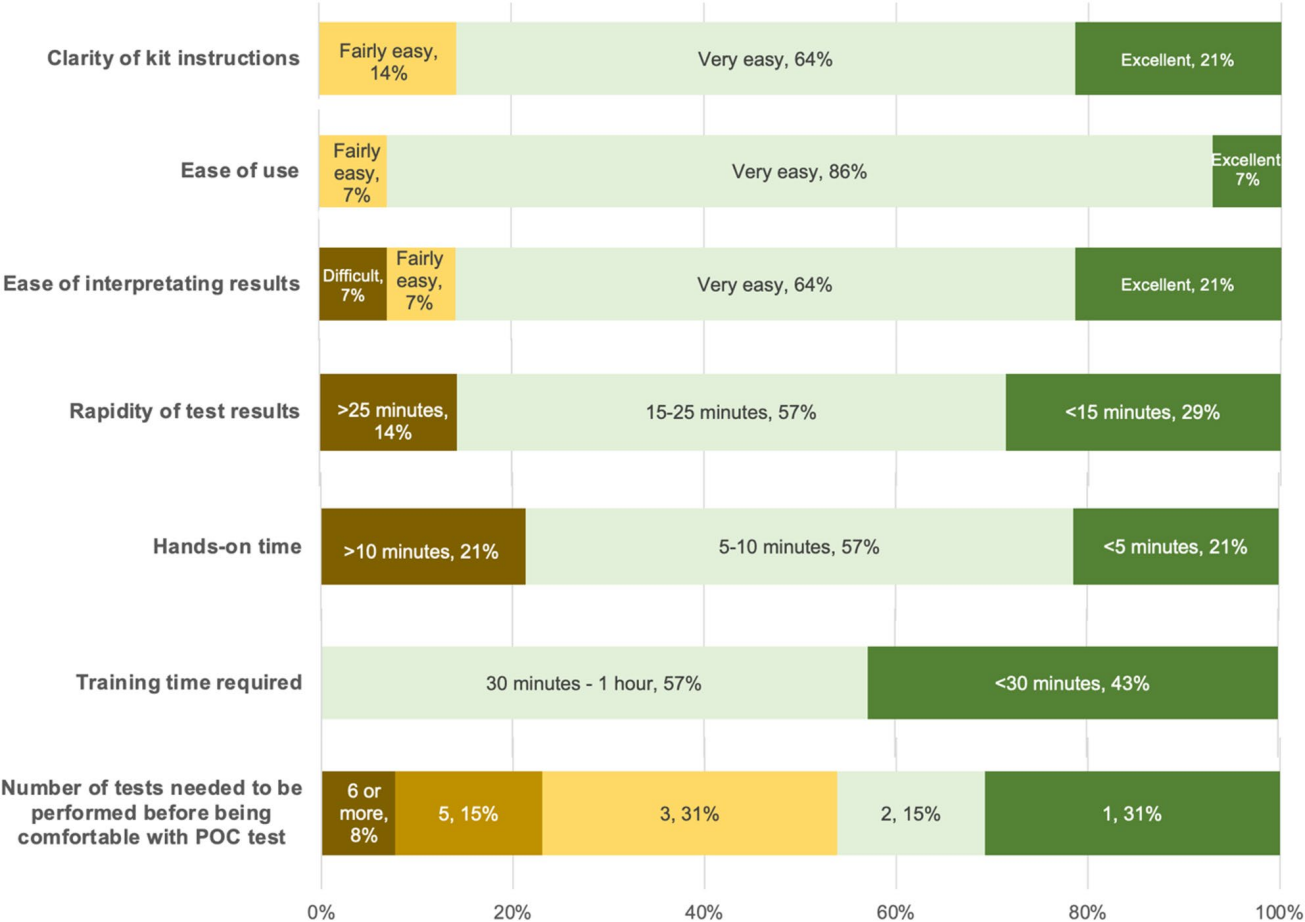
Operational characteristics of the OSOM^®^ BVBlue^®^ assessed by study staff (*N* = 14)

Nearly all women, 97.9% (978/999), stated they would be willing to wait up to 20 minutes for a POC test result, and 59.2% of women (591/999) up to 30 minutes. Figure 4 contains the results of the study staff questionnaire regarding the operational characteristics of OSOM^®^ BVBlue^®^. Of the 14 study staff members involved, all found the POC test easy to use and instructions clear, 13 felt the results were easy to interpret, and 12 reported test results were available within 25 minutes.

## Discussion

In this ANC setting in Zambia, OSOM^®^ BVBlue^®^ demonstrated sub-optimal sensitivity at 41.3%, and moderate-to-high specificity at 93.1%. Overall sensitivity in this study was at the lower end of the range compared to past studies that also used Nugent score as the reference standard (37–100%), whereas the specificity fell within the reported range (92.1–100%) [29–37] (Additional file 1 Table S1). Past studies targeted women with symptoms or risk factors and who were predominantly not pregnant, whereas our study uniquely evaluated the POC test as a general screening tool for pregnant women. Diagnostic accuracy of the POC test did not differ between symptomatic and asymptomatic participants. However, sensitivity varied significantly across different health centres, from 22.2% in Kabuta to 71.0% in Kafutuma. Kafutuma exhibited highest sensitivity at 71%, but it had the lowest specificity (88.3%) among the four health centres. In contrast, Kabuta had the highest specificity (95.1%) and lowest sensitivity (22.2%) among the centres. This suggests that Kafutuma tended to interpret POC test results more often as positive, whereas Kabuta tended to interpret them as negative. Indeed, the overall POC test positivity in Kafutuma was 24%, compared to 9% in Kabuta, despite Kabuta having a higher prevalence of BV as determined by Nugent score (Kafutuma: 22%; Kabuta: 26%). The interpretation of the colour of the liquid in the testing vessel could be influenced by factors such as room lighting or background colour. Study enrolment and testing were typically conducted in the morning, during which time the health centres relied on natural light from windows. Another potential explanation could be variation in clinical staff practices. However, there is no evidence to support this, as all staff members underwent identical instructions and collective training prior to the commencement of the study. Additionally, a single coordinator supervised the enrolment process each day across all four health centres. It is important to note that both Kabuta and Kafutuma had a relatively small number of participants (Kabuta:138, Kafutuma: 143), leading to greater uncertainty and wider confidence intervals.

Although the POC test sensitivity was sub-optimal, the functional information provided by this test still holds value. BV is characterised by a disturbance of the normally *Lactobacilli*-dominated vaginal flora, but its precise aetiology of causing adverse pregnancy and birth outcomes remains unknown, and current BV diagnosis based on morphological aspects might not capture the full picture. Sialidase, an enzyme produced by *Gardnerella* and some additional anaerobic bacteria, whose activity the OSOM^®^ BVBlue^®^ targets, cleaves terminal sialic acid residues off of vaginal mucosal glycans and might play a role in the attachment and colonisation of vaginal pathogens [38]. Sialidase is also shown to impair local immune defences, as indicated by its association with increased vaginal IL-1β and inhibition of IL8 and neutrophils [39]. A clinical study showed that among those with BV diagnosed by Nugent score, sialidase levels in early gestation correlated with the risk of preterm birth and late miscarriages [38]. Therefore, sialidase has a potential to be used as a biomarker of BV and its associated adverse pregnancy outcomes. Our study found a relatively high specificity (93.1%), with most false positive results corresponding to intermediate Nugent scores (4–6) (47/53). For diagnosing intermediate or higher Nugent score, the POC test was 98.7% sensitive.

From an operational standpoint, the enzyme-based POC test for BV was user-friendly, requiring minimal training and delivering results within 25 minutes in the majority of cases. However, while 85% (12 of 14) of study staff rated the ease of interpreting the POC test results as ‘very easy’ or ‘excellent’, one staff member (7%) found it ‘difficult,’ and another described it as ‘fairly easy.’ While OSOM^®^ BVBlue^®^ is a qualitative test, we observed a spectrum of colour changes from yellow (negative) to green and blue (positive). Some results were clearly blue, while others were greenish-yellow, making some diagnoses difficult. Additional file 1 Figure S2 contains photos to illustrate the range of intermediate colours among false positive cases.

The requirement of maintaining OSOM^®^ BVBlue^®^ kits in refrigerated conditions from 2° to 8 °C proved to be costly and operationally difficult in rural Zambia. An additional challenge was the relatively short shelf-life (six months) of the OSOM^®^ BVBlue^®^ kits. This was manageable in our study setting, but could prove difficult if deployed as part of routine ANC, necessitating frequent shipments with the risk of stockouts or increased waste. Addressing these challenges will be crucial for deployment of enzyme-based POC tests in resource-limited rural settings. These challenges may be less of an issue if the kits are used in resource-limited urban areas.

To the best of our knowledge, there are no Target Product Profiles (TPPs) for POC tests to diagnose BV. Findings from this study are valuable for development of minimal and optimal product profiles for POC test for BV. The development of these profiles, in turn, may support further research and development of POC tests for this highly prevalent condition and its important sexual and reproductive health sequelae.

## Conclusion

OSOM^®^ BVBlue^®^ showed sub-optimal sensitivity but relatively high specificity. Logistical challenges due to cold-chain requirements and short shelf-life need to be addressed for its effective routine use in resource-limited rural settings. The findings from this study are vital to the further development of POC tests that target BV.

## Supplementary Information

Below is the link to the electronic supplementary material.

Supplementary Material 1

## Data Availability

The datasets used and/or analysed during the current study are available from the corresponding author on reasonable request.

## Declarations

### About this supplement

This article has been published as part of BMC Infectious Diseases Volume 24 Supplement 1, 2024: Point-of-care Testing for Sexually Transmitted Infections: results of an independent multi-country clinic-based and clinic-utility evaluation of STI diagnostics (PRoSPeRo project). The full contents of the supplement are available online at https://bmcinfectdis.biomedcentral.com/articles/supplements/volume-24-supplement-1.

## Abbreviations

ANC: Antenatal care
BV: Bacterial vaginosis
CI: Confidence interval
IQR: Interquartile range
LR: Likelihood ratio
NAAT: Nucleic acid amplification test
NPV: Negative predictive value
OR: Odds ratio
POC: Point-of-care
PPV: Positive predictive value
ProSPeRo: Project on Sexually Transmitted Infection Point-of-care Testing
RR: Risk ratio
STI: Sexually transmitted infection
TV: Trichomonas vaginalis
WHO: World Health Organization

## References

[1] Redelinghuys MJ, et al. A cross-sectional study on the relationship of age, gestational age and HIV infection to bacterial vaginosis and genital Mycoplasma infection. BMJ Open. 2015;5:e008530. 26482771 10.1136/bmjopen-2015-008530 PMC4611850

[2] Jayaram PM, Mohan MK, Konje J. Bacterial vaginosis in pregnancy – a storm in the cup of tea. Eur J Obstet Gynecol Reprod Biol. 2020;253:220–4. 32889328 10.1016/j.ejogrb.2020.08.009

[3] Park FJ, Rosca AS, Chico RM. Prevalence of bacterial vaginosis among pregnant women attending antenatal care in low - and middle ‐ income countries between 2000 and 2020: a systematic review and meta ‐ analysis. 2024;1–11. 10.1002/rfc2.99.

[4] Leitich H, et al. Bacterial vaginosis as a risk factor for preterm delivery: A meta-analysis. Am J Obstet Gynecol. 2003;189:139–47. 12861153 10.1067/mob.2003.339

[5] Acobsson BJ, Ernevi PP, Hidekel LC, J J. Bacterial vaginosis in early pregnancy May predispose for preterm birth and postpartum endometritis. Acta Obstet Gynecol Scand. 2002;81:1006–10. 12421167 10.1034/j.1600-0412.2002.811103.x

[6] Bautista CT, et al. Association of bacterial vaginosis with chlamydia and gonorrhea among women in the U.S. Army. Am J Prev Med. 2017;52:632–9. 27816380 10.1016/j.amepre.2016.09.016

[7] Atashili J, Poole C, Ndumbe PM, Adimora AA, Smith JS. Bacterial vaginosis and HIV acquisition: A meta-analysis of published studies. Aids. 2008;22:1493–501. 18614873 10.1097/QAD.0b013e3283021a37 PMC2788489

[8] Gillet E, et al. Bacterial vaginosis is associated with uterine cervical human papillomavirus infection: a meta-analysis. BMC Infect Dis. 2011;11. 10.1186/1471-2334-11-10 PMC3023697 21223574

[9] Gillet E, et al. Association between bacterial vaginosis and cervical intraepithelial neoplasia: systematic review and meta-analysis. PLoS One. 2012;7. 10.1371/journal.pone.0045201 PMC3462776 23056195

[10] Dahoud W, Michael CW, Gokozan H, Nakanishi AK, Harbhajanka A. Association of bacterial vaginosis and human papilloma virus infection with cervical squamous intraepithelial lesions. Am J Clin Pathol. 2019;152:185–9. 31065675 10.1093/ajcp/aqz021

[11] Rebouças KF, et al. Treatment of bacterial vaginosis before 28 weeks of pregnancy to reduce the incidence of preterm labor. Int J Gynecol Obstet. 2019;146:271–6. 10.1002/ijgo.12829 31022300

[12] Ugwumadu A, Manyonda I, Reid F, Hay P. Effect of early oral clindamycin on late miscarriage and preterm delivery in asymptomatic women with abnormal vaginal flora and bacterial vaginosis: A randomised controlled trial. Lancet. 2003;361:983–8. 12660054 10.1016/S0140-6736(03)12823-1

[13] Thinkhamrop J, Hofmeyr GJ, Adetoro O, Lumbiganon P, Ota E. Antibiotic prophylaxis during the second and third trimester to reduce adverse pregnancy outcomes and morbidity. Cochrane Database Syst Rev. 2015;6:CD002250. 10.1002/14651858.CD002250.pub3 PMC7154219 26092137

[14] World Health Organization. Bacterial vaginosis. 2023. https://www.who.int/news-room/fact-sheets/detail/bacterial-vaginosis. Accessed 10 Dec 2025.

[15] World Health Organization. Laboratory and point-of-care diagnostic testing for sexually transmitted infections, including HIV. 2023. https://www.who.int/publications/i/item/9789240077089. Accessed 10 Dec 2025.

[16] World Health Organization. Guidelines for the management of symptomatic sexually transmitted infections. 2021. https://iris.who.int/bitstream/handle/10665/342523/9789240024168-eng.pdf. Accessed 10 Dec 2025. 34370424

[17] Tann CJ, et al. Lack of effectiveness of syndromic management in targeting vaginal infections in pregnancy in entebbe. Uganda Sex Transm Infect. 2006;82:285–9. 16877576 10.1136/sti.2005.014845 PMC2564710

[18] Romoren M, et al. Trichomoniasis and bacterial vaginosis in pregnancy: inadequately managed with the syndromic approach. Bull World Health Organ. 2007;85:297–304. 17546311 10.2471/BLT.06.031922 PMC2636319

[19] Chaponda EB, Bruce J, Michelo C, Chandramohan D, Chico RM. Assessment of syndromic management of curable sexually transmitted and reproductive tract infections among pregnant women: an observational cross- sectional study. BMC Pregnancy and Childbirth 2021;21. 10.1186/s12884-021-03573-3 PMC7847014 33516183

[20] Bossuyt PM, Reitsma JB, Bruns DE, Gatsonis CA, Glasziou PP, Irwig L, LijmerJG Moher D, Rennie D, de Vet HCW, Kressel HY, Rifai N, Golub RM, Altman DG, Hooft L, Korevaar DA, Cohen JF. F. the S. G. STARD2015: an updated list of essential items for reporting diagnostic accuracy studies. 2015. https://www.equator-network.org/reporting-guidelines/stard/. Accessed 10 Dec 2025.

[21] Cordioli M, et al. Standardised protocol for a prospective international multicentre clinical-based evaluation of point-of-care tests for the screening of genital and extragenital chlamydial and gonococcal infections in men who have sex with men and for the screening of genital chlamydial, gonococcal and *Trichomonas vaginalis* infections in at risk women. BMJ Open. 2024;14:e073565. 38885995 10.1136/bmjopen-2023-073565 PMC11184175

[22] Zambia Statistics Agency. 2022 Census of population and housing. 2022. https://www.zamstats.gov.zm/2022-census. Accessed 10 Dec 2025.

[23] Harris PA, et al. Research electronic data capture (REDCap)—A metadata-driven methodology and workflow process for providing translational research informatics support. J Biomed Inf. 2009;42:377–81. 10.1016/j.jbi.2008.08.010 PMC2700030 18929686

[24] Reitsma JB, et al. Bivariate analysis of sensitivity and specificity produces informative summary measures in diagnostic reviews. J Clin Epidemiol. 2005;58:982–90. 16168343 10.1016/j.jclinepi.2005.02.022

[25] Dwamena B. MIDAS: Stata module for meta-analytical integration of diagnostic test accuracy studies. Stat Softw Compon. 2007;1–2.

[26] Nyaga VN, Arbyn M. Metadta: a Stata command for meta-analysis and meta-regression of diagnostic test accuracy data – a tutorial. Arch Public Heal. 2022;80:1–15. 10.1186/s13690-021-00747-5 PMC8962039 35351195

[27] Nyaga VN, Arbyn M. Comparison and validation of Metadta for meta-analysis of diagnostic test accuracy studies. Res Synth Methods. 2023;14:544–62. 36999350 10.1002/jrsm.1634

[28] StataCorp. Stata statistical software: release 18. College station. TX: StataCorp LLC; 2023.

[29] Khatoon R, Ahmad S, Jahan N. OSOM BV blue test: A new point-of-care test for diagnosing bacterial vaginosis and its comparison with gram staining. Afr J Microbiol Res. 2013;7:4103–6.

[30] Khatoon R, Jaha N, Ahmad S, Rabbani T. Comparison of OSOM BV blue test with conventional methods for diagnosis of bacterial vaginosis. Afr J Microbiol Res. 2013;7:3698–703. https://www.mtnstopshiv.org/sites/default/files/attachments/Poster. Rabe final 6-23-09.pdf.

[31] Shujatullah F, Khan HM, Khatoon R, Rabbani T, Malik A. An evaluation of OSOM BV blue test in the diagnosis of bacterial vaginosis. Asian Pac J Trop Med. 2010;3:574–6.

[32] Haamid FW, Holland-Hall C, Berry J, Leber AL. Evaluation of the OSOM BVBlue test for diagnosis of bacterial vaginosis in an Adolescent, young adult population. J Adolesc Heal. 2014;54:S49–50.

[33] Rabe L. The sensitivity and specificity of OSOM^®^ Rapid Trichomonas vaginalis and Bacterial Vaginosis (BVBlue^®^) tests. Poster presented at: Magee-Womens Research Institute. Avaiable from: https://mtnstopshiv.org/sites/default/files/attachments/Poster%20Rabe%20final%206-23-09.pdf. Accessed 31 Oct 2022

[34] Madhivanan P, et al. Performance of BVBlue rapid test in detecting bacterial vaginosis among women in Mysore, India. Infect Dis Obstet Gynecol. 2014;2014. 10.1155/2014/908313 PMC3913452 24526829

[35] Kampan NC, et al. Evaluation of BV ^®^ blue test kit for the diagnosis of bacterial vaginosis. Sex Reprod Healthc. 2011;2:1–5. 21147452 10.1016/j.srhc.2010.11.002

[36] Myziuk L, Romanowski B, Johnson SC. BVBlue test for diagnosis of bacterial vaginosis. J Clin Microbiol. 2003;41:1925–8. 12734228 10.1128/JCM.41.5.1925-1928.2003 PMC154737

[37] Intra J, et al. Rapid detection of Sialidase activity for the diagnosis of bacterial vaginosis. Int J Curr Microbiol Appl Sci. 2018;7:3898–908.

[38] Cauci S, Culhane JF. High sialidase levels increase preterm birth risk among women who are bacterial vaginosispositive in early gestation. Am J Obstet Gynecol. 2011;204:142.e1-142.e9. 10.1016/j.ajog.2010.08.061 21055720

[39] Cauci S, Culhane JF, Di Santolo M, McCollum K. Among pregnant women with bacterial vaginosis, the hydrolytic enzymes sialidase and prolidase are positively associated with interleukin-1β. Am J Obstet Gynecol. 2008;198:132.e1-132.e7. 10.1016/j.ajog.2007.05.035 17714681

