# Supplementary material for: Diagnostic accuracy of an enzyme-based point-of-care test versus Nugent score for bacterial vaginosis among pregnant women attending routine antenatal care in Zambia
Source: BMC Infect Dis. 2026 Jan 31;24(Suppl 1):1483. doi: 10.1186/s12879-026-12714-y (PMC12862899; doi:10.1186/s12879-026-12714-y)
Supplement: Supplementary file 1 — Supplementary Material 1 [file 12879_2026_12714_MOESM1_ESM.docx]

**Supplementary material**

**Main article**

Sorano S, Chaponda EB, Mirandola M, Chikwanda E, Mwewa V, Mulenga JM, Chaponda M, Ghilardi L, Harding-Esch EM, Smith C, Matsui M, Chandramohan D, Ali MM, Blondeel K, Unemo M, Toskin I, Chico RM; Diagnostic accuracy of an enzyme-based point-of-care test versus Nugent scoring for bacterial vaginosis among pregnant women attending routine antenatal care in Zambia; *BMC Infect Dis*.

**Contents**

[**Figure S1**](#_Figure_S1._Fagan’s)**.** Fagan’s nomogram demonstrating diagnostic utility of OSOM® BVBlue®︎ point-of-care test among pregnant women attending antenatal care in Nchelenge, Zambia

[**Figure S2**](#_Figure_S2._Photos)**.** Photos of OSOM® BVBlue® in false positive cases

[**Table S1**](#_Table_S1._Published)**.** Published studies of OSOM® BVBlue®︎ point-of-care test among women which use Nugent score for reference standard

[**Table S2**](#_Table_S2_Results)**.** Results of OSOM® BVBlue® and Nugent scoring for bacterial vaginosis in pregnant women by site in Nchelenge, Zambia

[**Case Report Form**](#_Case_Report_Form)

[**Provider Questionnaire Form**](#_Provider_Questionnaire_Form)

# **Figure S1.** Fagan’s nomogram demonstrating diagnostic utility of OSOM® BVBlue®︎ point-of-care test among pregnant women attending antenatal care in Nchelenge, Zambia

1. Overall participants (both women with and without BV-associated symptoms)
2. Asymptomatic participants (women without BV-associated symptoms)
3. Symptomatic participants (women with BV-associated symptoms)

Note: BV associated symptoms are: unusual vaginal discharge, pain during urination, itching or burning of the vulva, Prob: probability, Pos: positive, Neg: negative, LR: likelihood ratio

# **Figure S2.** Photos of OSOM® BVBlue® in false positive cases

| 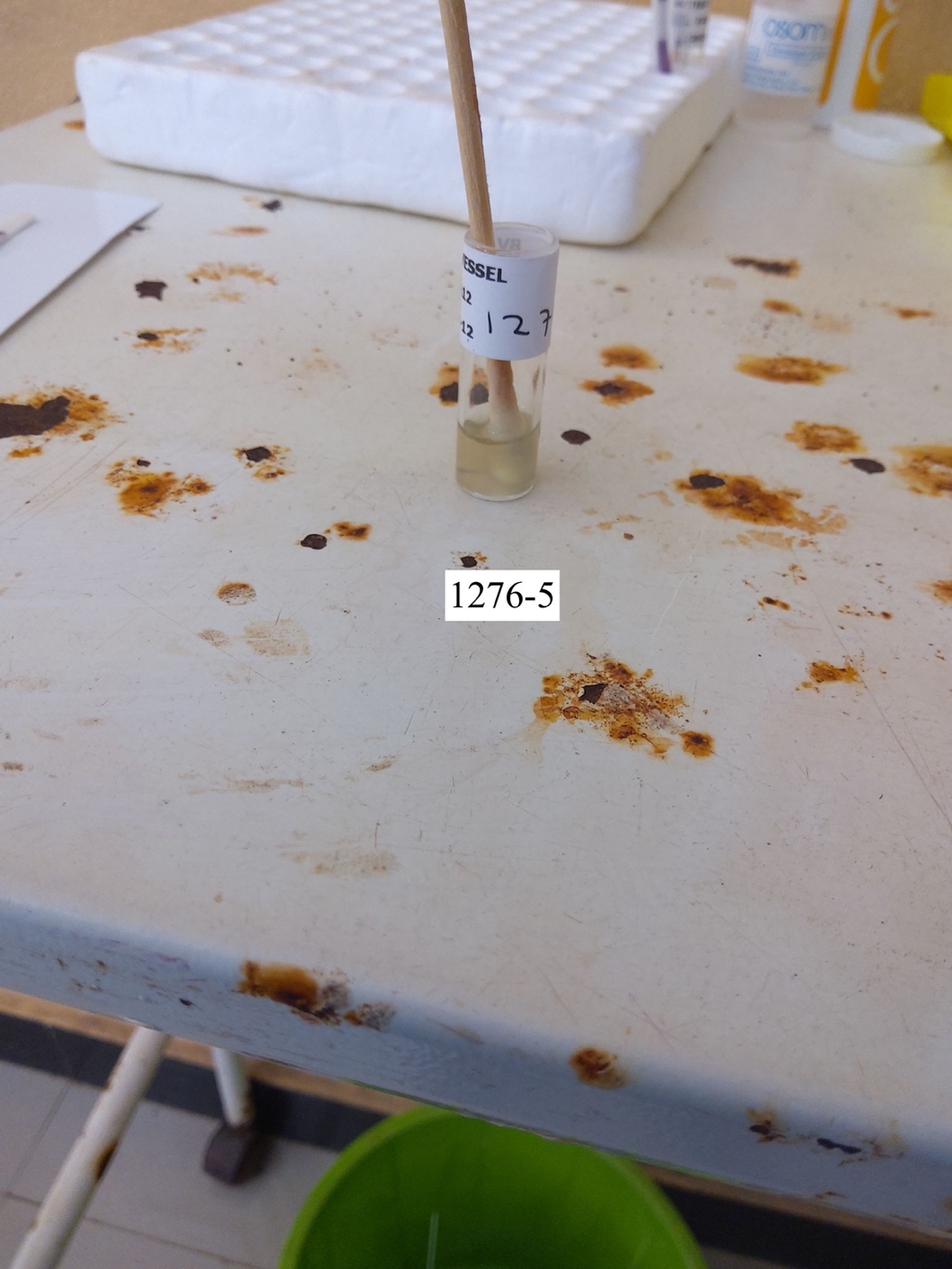 | 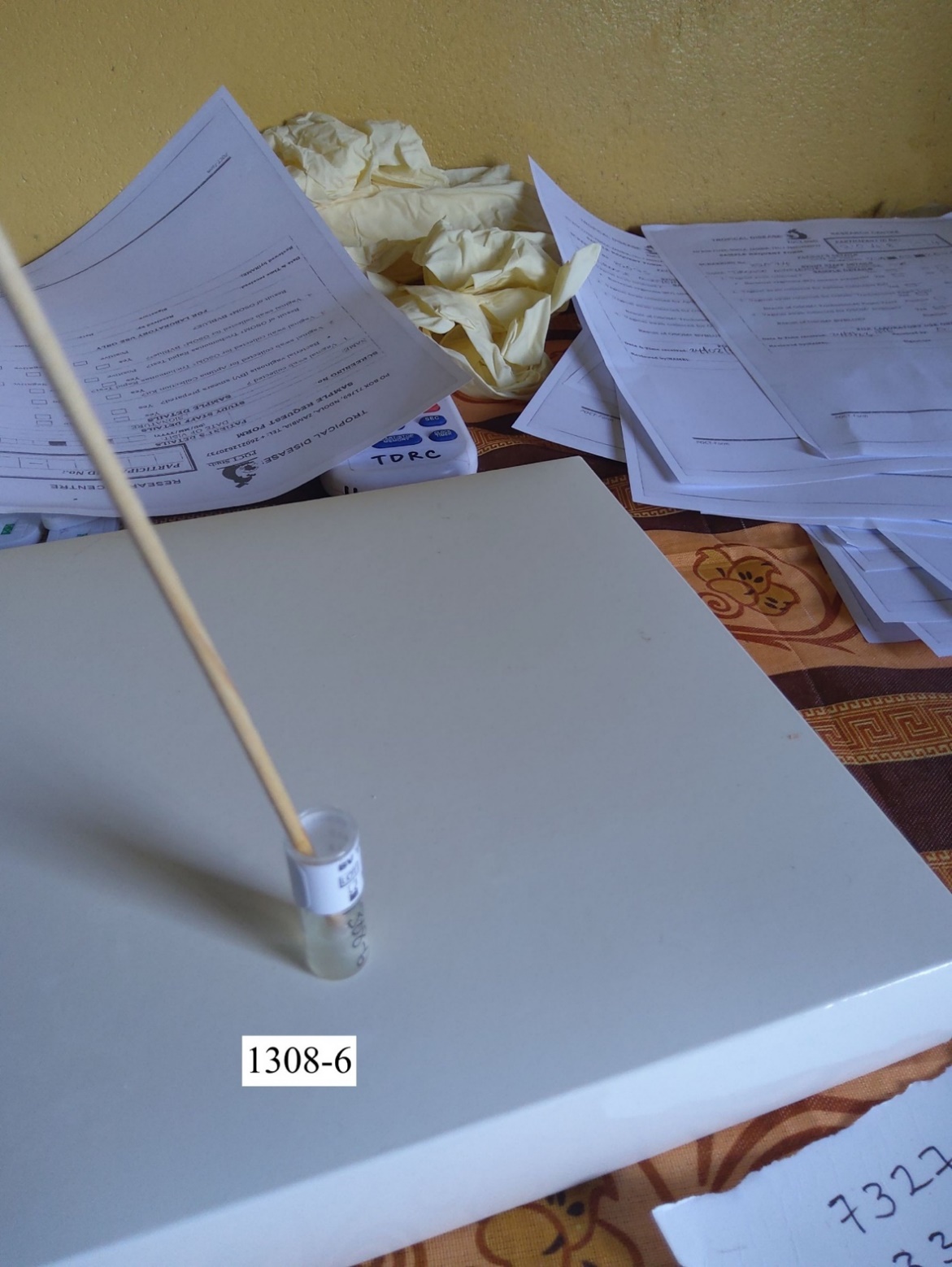 | 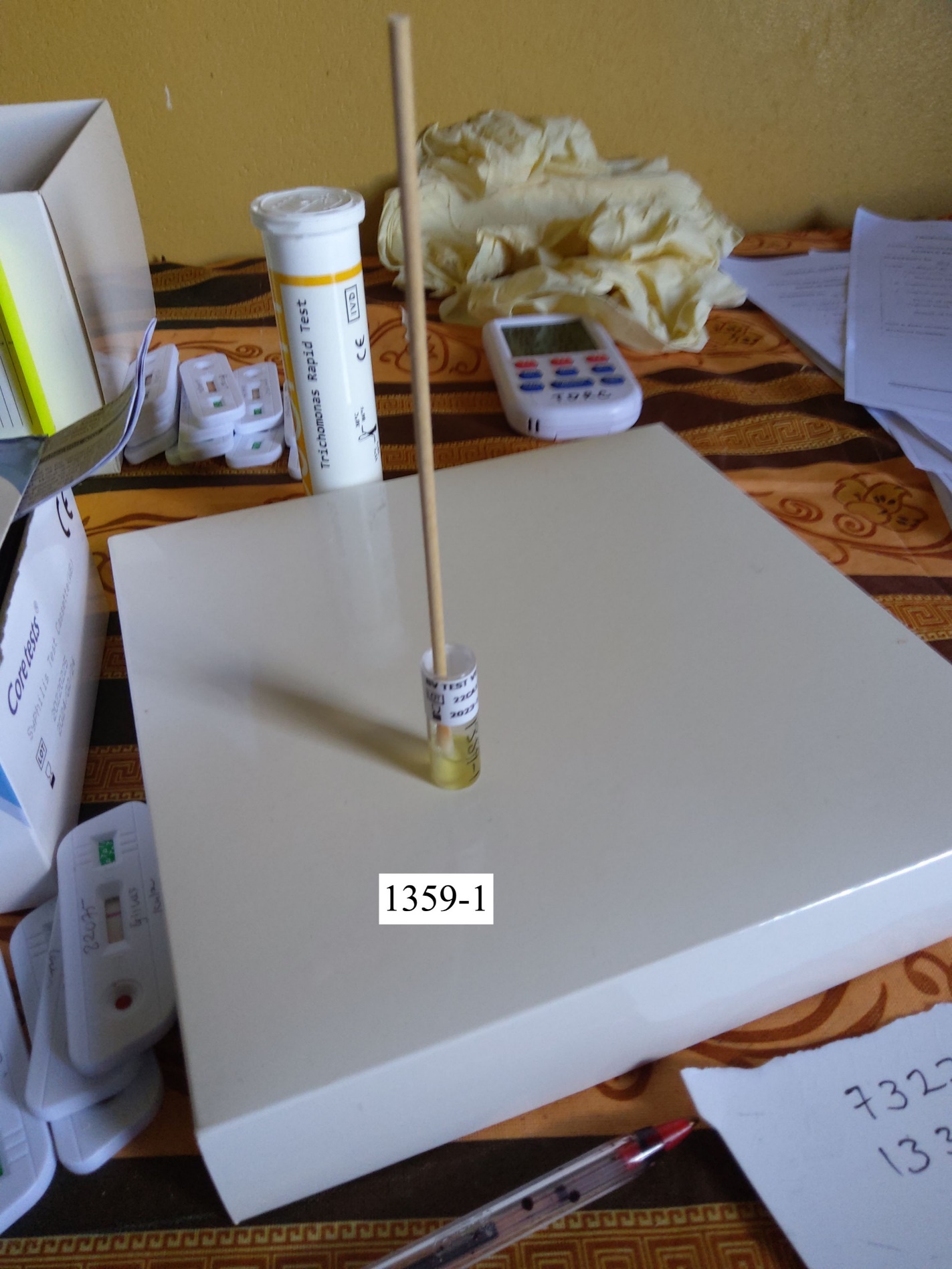 |
| --- | --- | --- |
| 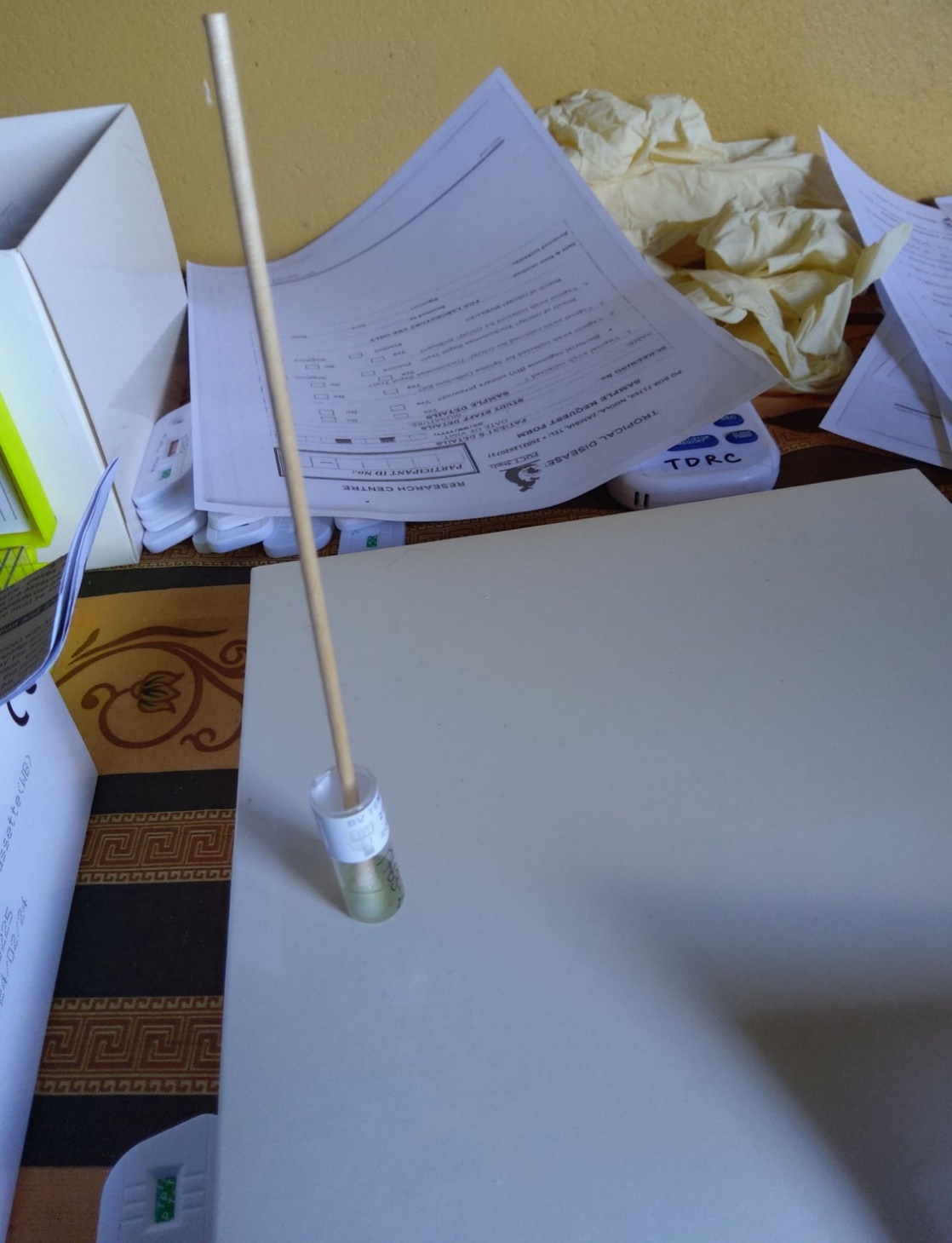 | 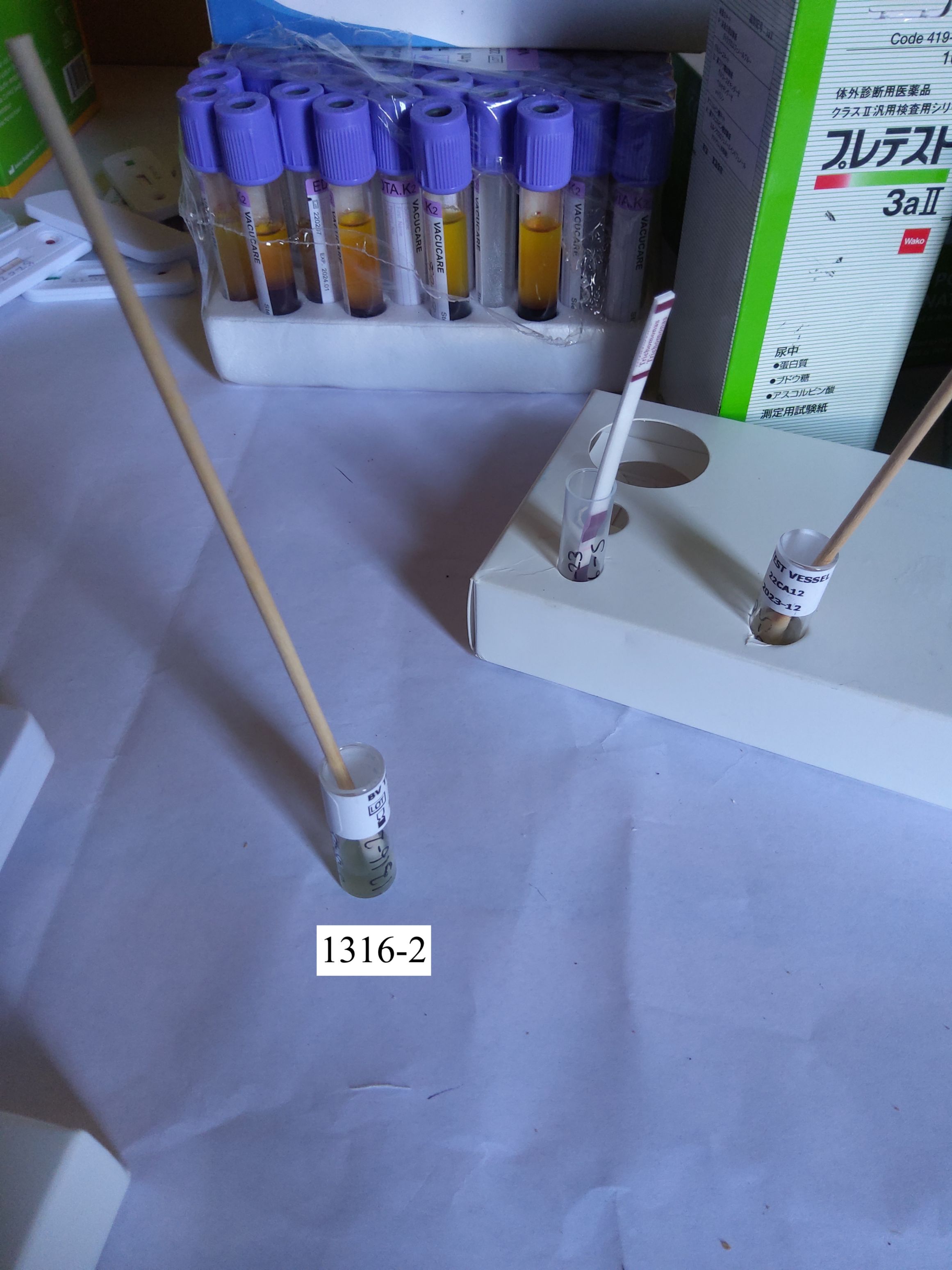 | 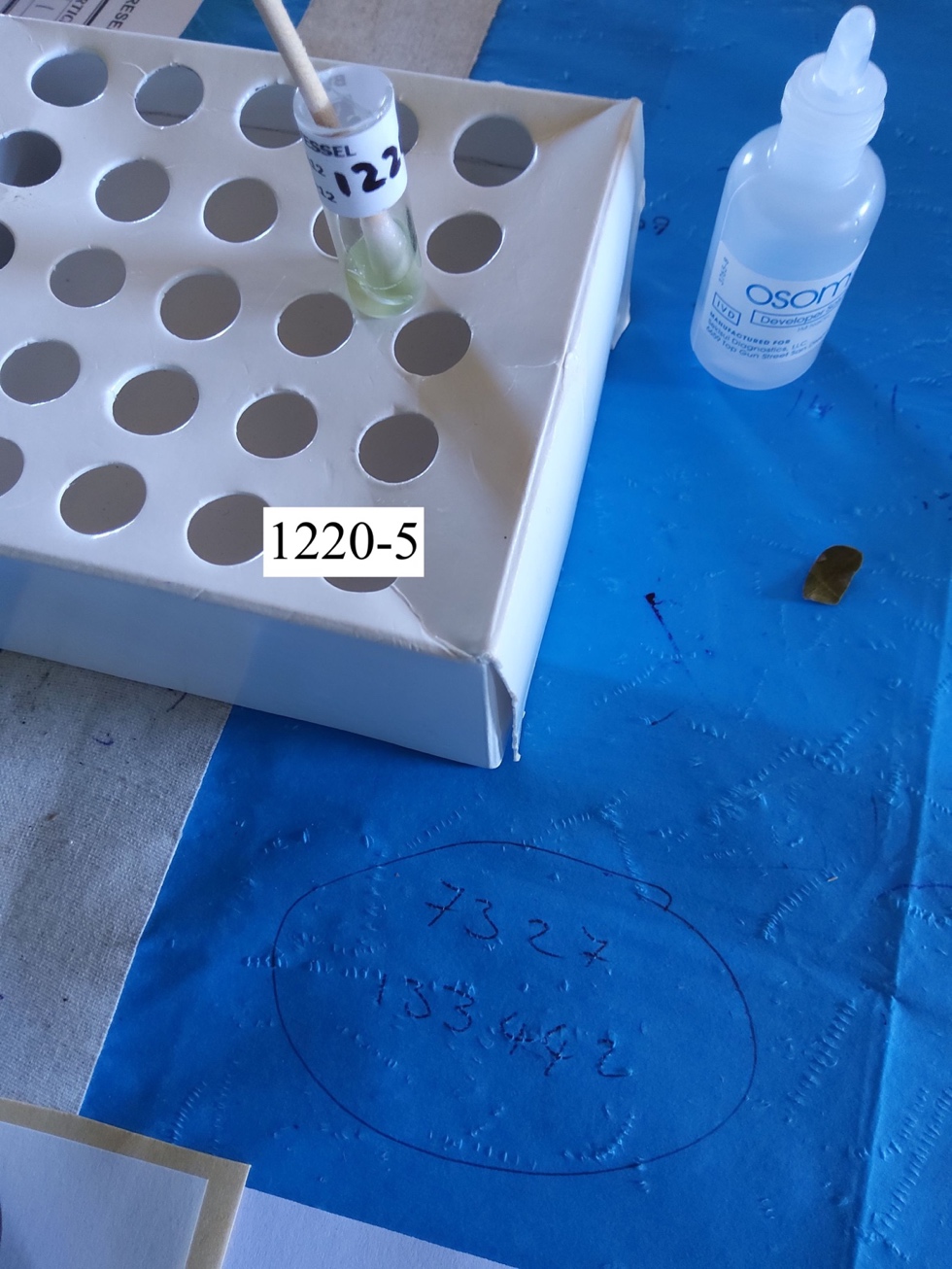 |
| 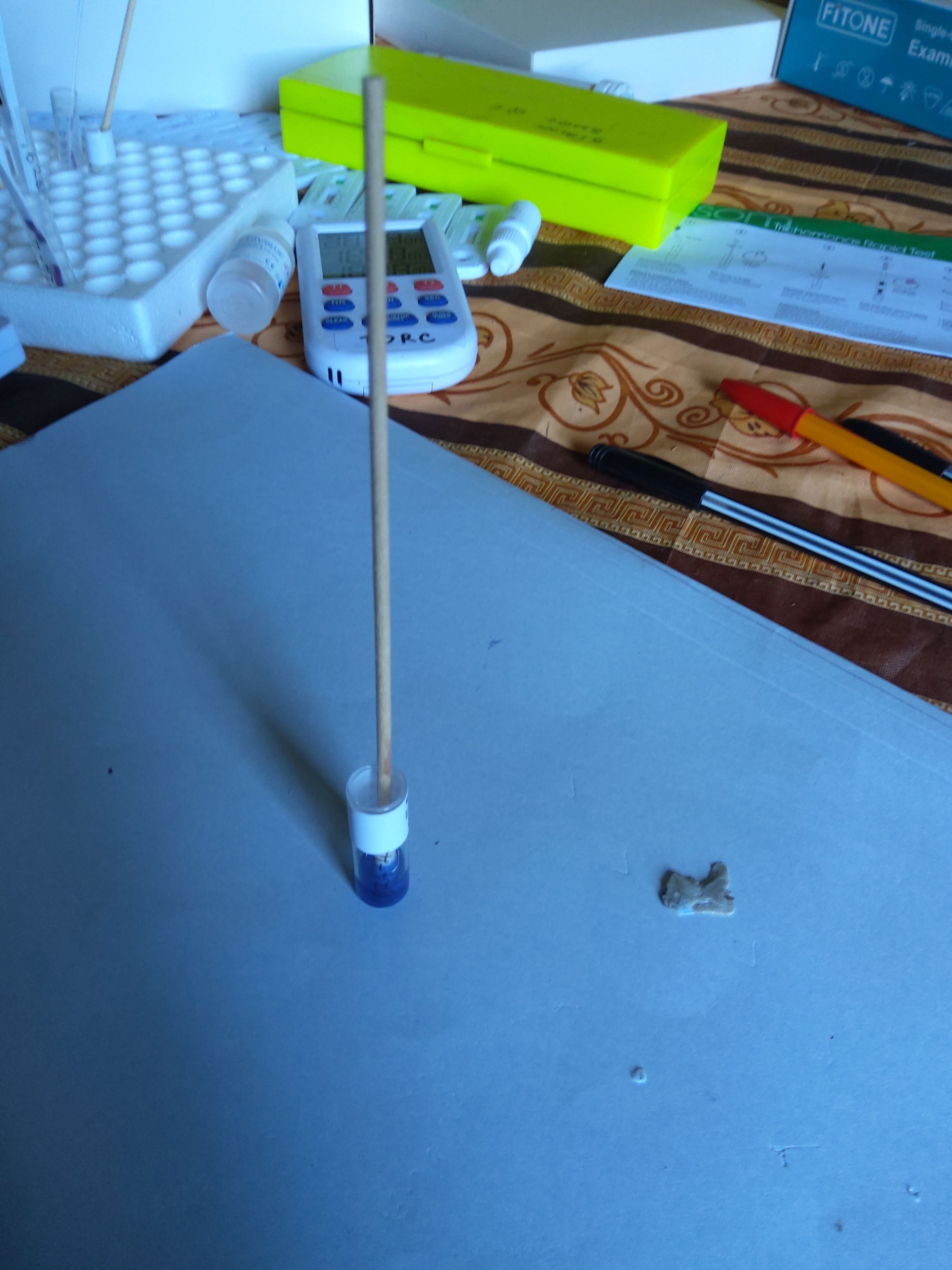 | 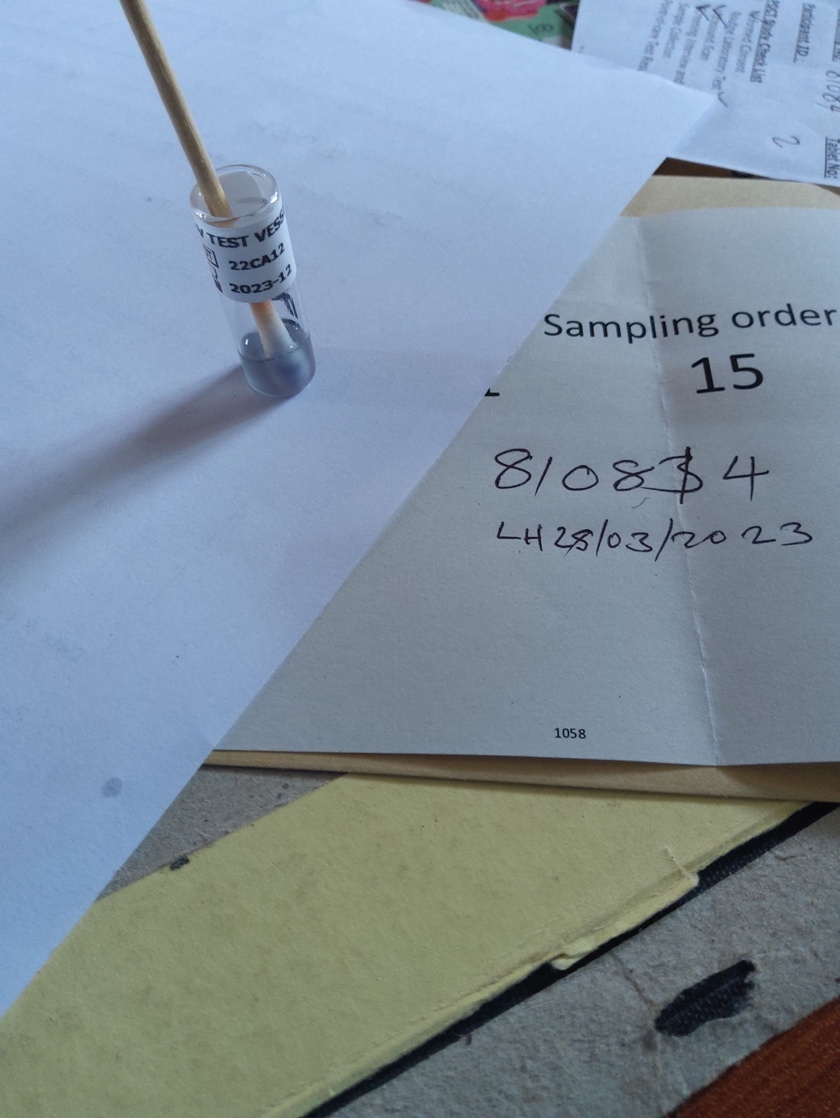 | 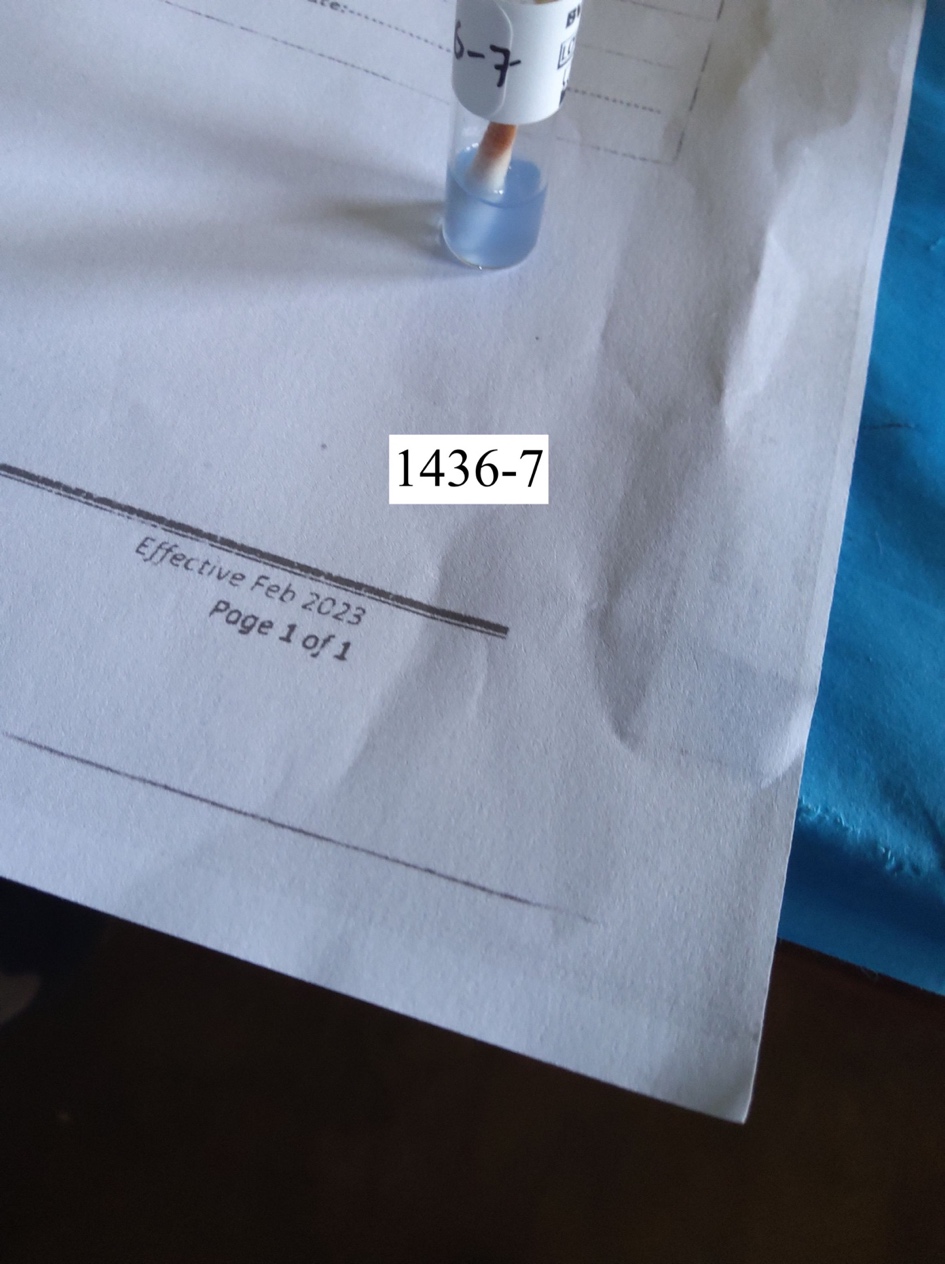 |
| 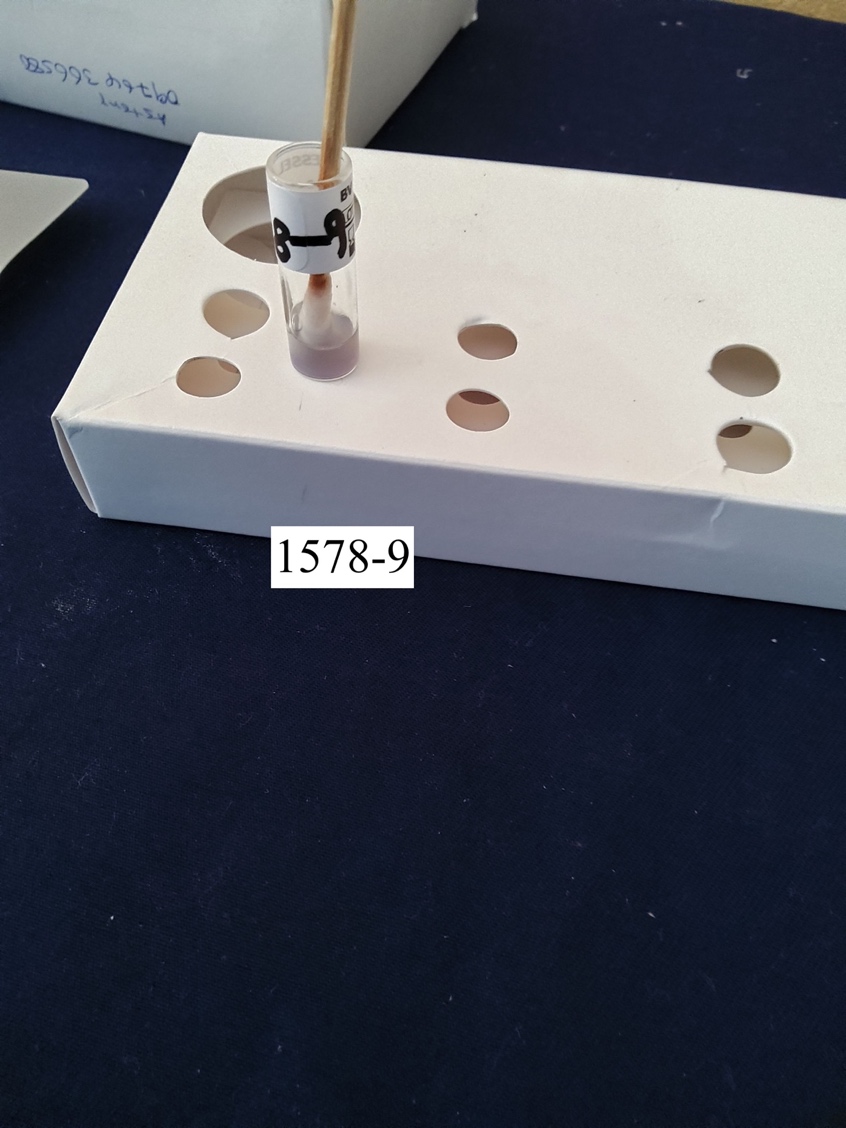 | 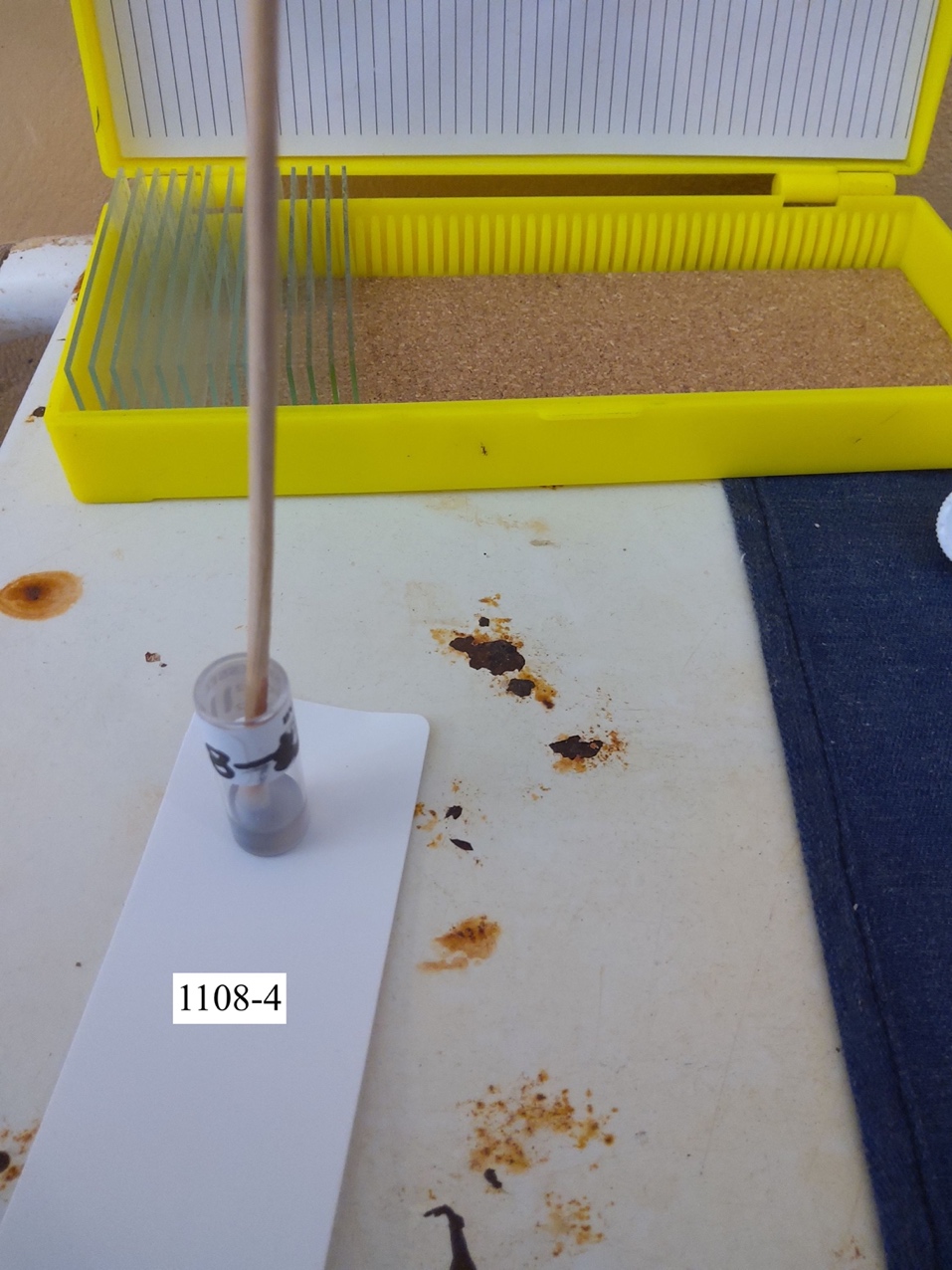 | 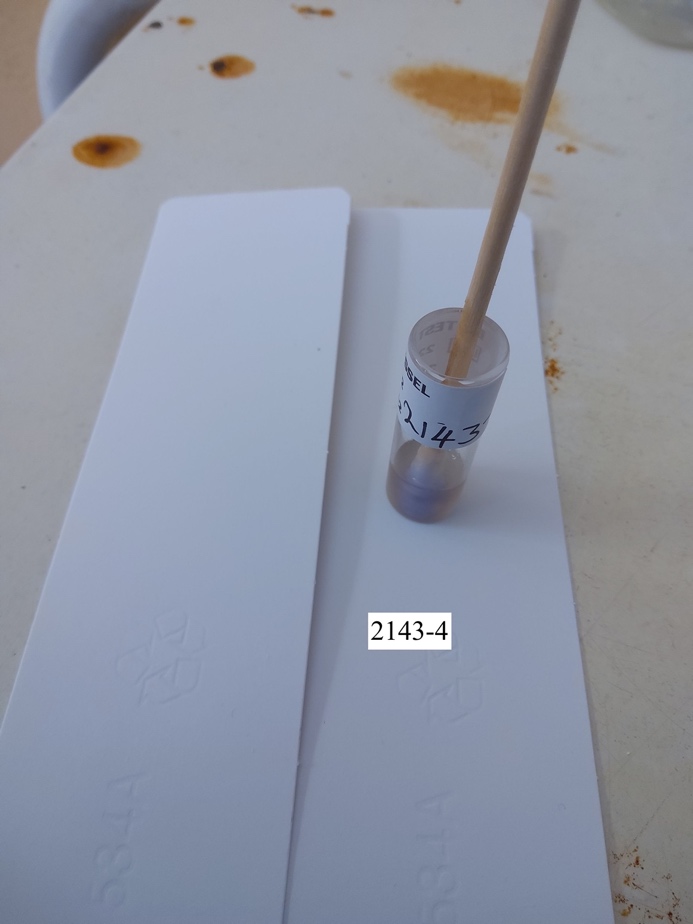 |

# **Table S1.** Published studies of OSOM® BVBlue®︎ point-of-care test among women which use Nugent score for reference standard

| **Author/ publication year** | **Country** | **Year(s) of study** | **Sample size** | **Symptomatic** | **Pregnant** | **Prevalence (%)** | **Reference test** | **Sensitivity (%)** | **Specificity (%)** | **Sample**  **collection** |
| --- | --- | --- | --- | --- | --- | --- | --- | --- | --- | --- |
| Khatoon 2013 [29, 30] | India | NR | 635 | Yes | Both | 60.8 | Nugent score | 95.3 | 92.1 | Clinician |
| Shujatullah 2010 [31] | India | 2008-09 | 405 | Yes | Yes: 114  No: 291 | 60.7 | Nugent score | 97.6 | 97.5 | Clinician |
| Haamid 2014 [32] | USA | 2012-13 | 100 | Yes | No | 61.0 | Nugent score | 62.3 | 100 | Clinician |
| Rabe, NR [33] | USA | NR | 394 | Yes/No | No | 47.0 | Nugent score | 73 | 99 | Clinician |
|  |  |  | 190^*^ | Yes | No | NR |  | 77 | 100 |  |
|  |  |  | 204^*^ | No | No | NR |  | 69 | 98 |  |
| Madhivanan 2014 [34] | India | 2009-10 | 266 | Yes/No | No | 45.1 | Nugent score^†^ | 37 | 95 | Clinician |
|  |  |  | 156^*^ | Yes | No | NR |  | 38 | 95 |  |
| Kampan 2011 [35] | Malaysia | 2006-07 | 151 | Yes: 55.6%  No: 44.4% | No | 22.5 | Nugent score | 100 | 98.3 | Clinician |
| Myziuk 2003 [36] | USA | NR | 57 | Yes: 54%  No: 46% | No | 21.1 | Nugent score | 91.7 | 97.8 | Clinician |
| Intra 2018 [37] | Italy | 2016-17 | 352 | Yes | No | 16.5 | Nugent score | 39.7 | 94.9 | Clinician |

NR: not reported, OPD: outpatient department, ANC: antenatal clinic; ^*^190 and 204 are subgroups of 394 (Rabe) and 156 is a subgroup of 266; ^†^ Madhivanan 2014 excludes cases of intermediate Nugent score (4-6)

# **Table S2** Results of OSOM® BVBlue® and Nugent scoring for bacterial vaginosis in pregnant women by site in Nchelenge, Zambia

| **Site** | OSOM® BVBlue® **result** | **Nugent scoring** | | | |
| --- | --- | --- | --- | --- | --- |
|  | | **BV diagnosis**  (7-10 Nugent) | **Intermediate**  (4-6 Nugent) | **No BV**  (0-3 Nugent) | **Total** |
| **Kabuta** | | | | | |
|  | Positive | 8 | 4 | 1 | 13 |
|  | Negative | 28 | 33 | 64 | 125 |
|  | Indeterminate or invalid | 0 | 0 | 0 | 0 |
|  | Total | 36 | 37 | 64 | 138 |
| **Kafutuma** | | | | | |
|  | Positive | 22 | 10 | 3 | 35 |
|  | Negative | 9 | 39 | 59 | 107 |
|  | Indeterminate or invalid | 0 | 1 | 0 | 1 |
|  | Total | 31 | 50 | 62 | 143 |
| **Kashikishi** | | | | | |
|  | Positive | 37 | 20 | 2 | 59 |
|  | Negative | 60 | 124 | 193 | 377 |
|  | Indeterminate or invalid | 0 | 0 | 0 | 0 |
|  | Total | 97 | 144 | 195 | 436 |
| **Nchelenge** | | | | | |
|  | Positive | 25 | 13 | 0 | 38 |
|  | Negative | 42 | 72 | 130 | 244 |
|  | Indeterminate or invalid | 0 | 0 | 0 | 0 |
|  | Total | 67 | 85 | 130 | 282 |
| **All sites combined** | | | | | |
|  | Positive | 92 | 47 | 6 | 145 |
|  | Negative | 139 | 268 | 446 | 853 |
|  | Indeterminate or invalid | 0 | 1 | 0 | 1 |
|  | Total | 231 | 316 | 452 | 999 |

# **Case Report Form**

**
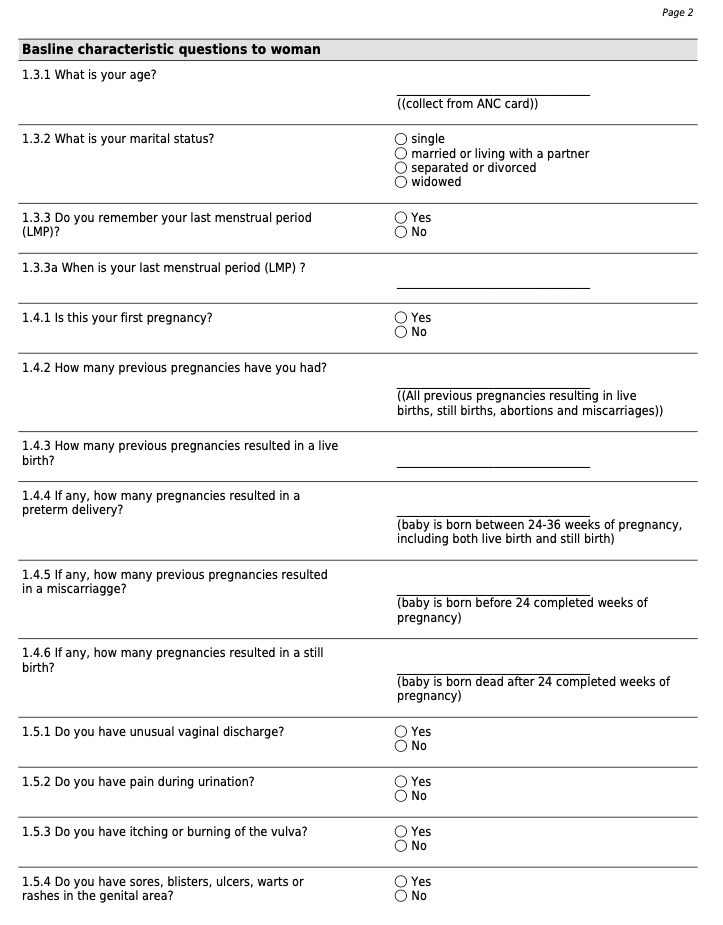
**

**
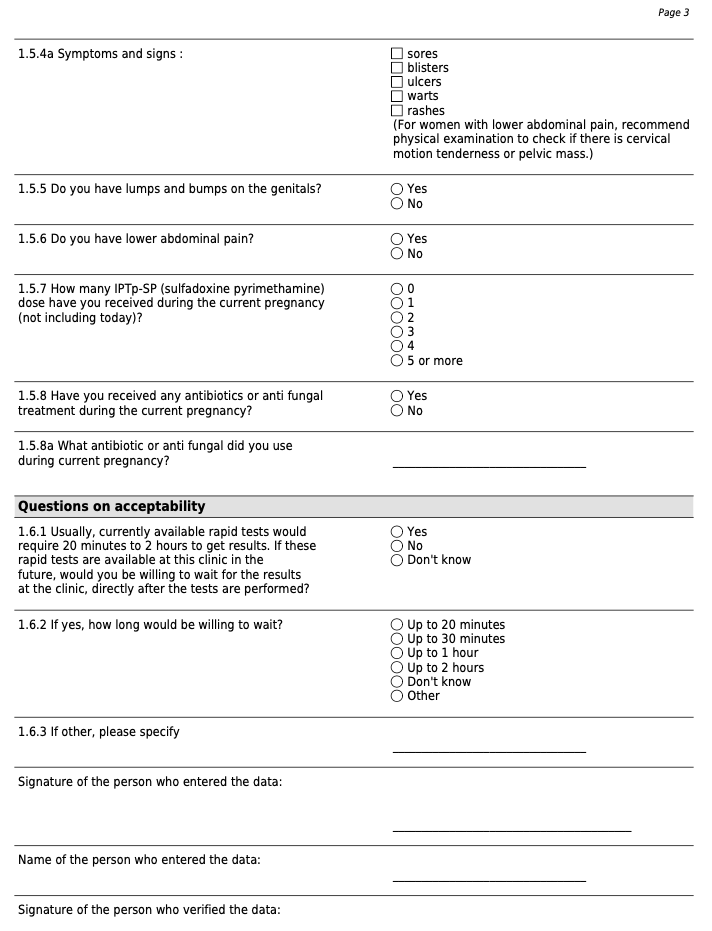
**

**
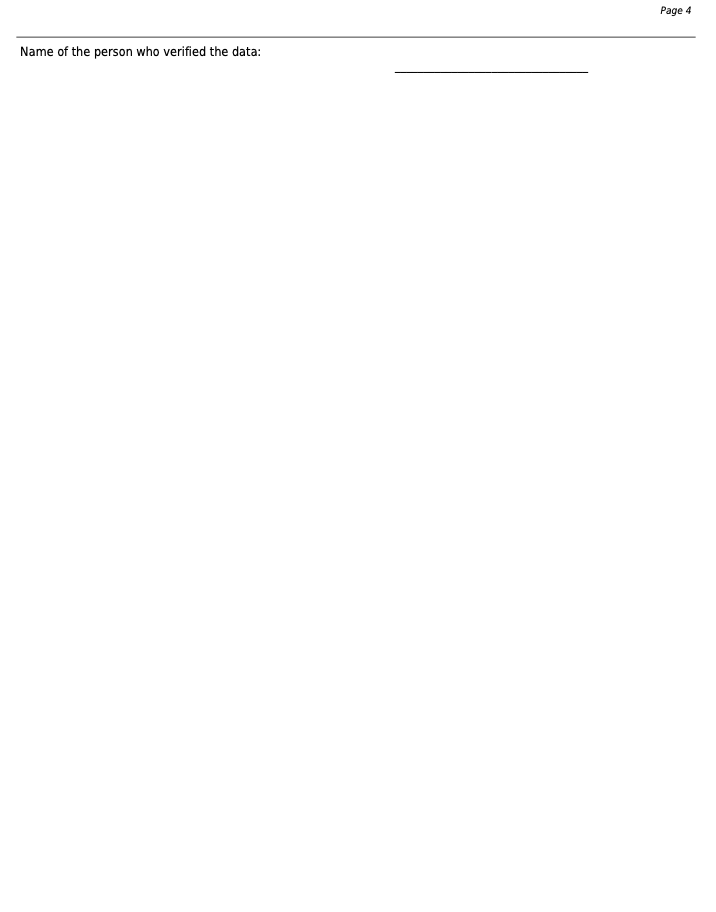
**

# **Provider Questionnaire Form**

**
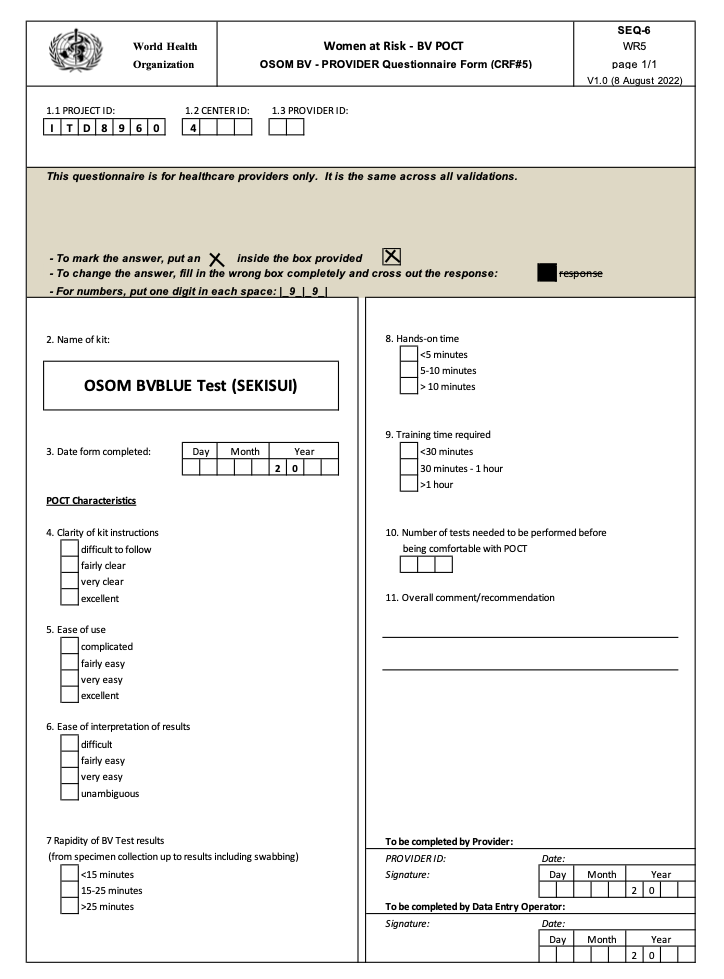
**
